# Computational evidence for nitro derivatives of quinoline and quinoline N-oxide as low-cost alternative for the treatment of SARS-CoV-2 infection

**DOI:** 10.1038/s41598-021-85280-9

**Published:** 2021-03-18

**Authors:** Letícia C. Assis, Alexandre A. de Castro, João P. A. de Jesus, Eugenie Nepovimova, Kamil Kuca, Teodorico C. Ramalho, Felipe A. La Porta

**Affiliations:** 1 Department of Chemistry, Federal University of Lavras, Lavras, Minas Gerais CEP 37200-000 Brazil; 2 Laboratório de Nanotecnologia E Química Computacional, Universidade Tecnológica Federal Do Paraná, Londrina, PR 86036-370 Brazil; 3 Department of Chemistry, Faculty of Science, University of Hradec Kralove, Rokitanskeho 62, 500 03 Hradec Králové, Czech Republic

**Keywords:** Computational biology and bioinformatics, Medical research, Molecular medicine, Chemistry

## Abstract

A new and more aggressive strain of coronavirus, known as SARS-CoV-2, which is highly contagious, has rapidly spread across the planet within a short period of time. Due to its high transmission rate and the significant time–space between infection and manifestation of symptoms, the WHO recently declared this a pandemic. Because of the exponentially growing number of new cases of both infections and deaths, development of new therapeutic options to help fight this pandemic is urgently needed. The target molecules of this study were the nitro derivatives of quinoline and quinoline N-oxide. Computational design at the DFT level, docking studies, and molecular dynamics methods as a well-reasoned strategy will aid in elucidating the fundamental physicochemical properties and molecular functions of a diversity of compounds, directly accelerating the process of discovering new drugs. In this study, we discovered isomers based on the nitro derivatives of quinoline and quinoline N-oxide, which are biologically active compounds and may be low-cost alternatives for the treatment of infections induced by SARS-CoV-2.

## Introduction

We are currently facing a new coronavirus disease designated as COVID-19. It started in China and has spread rapidly around the world, resulting in serious threats to international health and the economy^1,2^. The International Committee on the Taxonomy of Viruses denominated the virus as severe acute respiratory syndrome coronavirus 2 (SARS-CoV-2). This denomination is derived from the fact that the RNA genome is approximately 82% identical to the SARS coronavirus (SARS-CoV)^3^. In addition, the SARS-CoV-2 reveals a 79% similarity with SARS (Severe Acute Respiratory Syndrome) coronavirus and a 50% similarity with MERS (Middle Eastern Respiratory Syndrome) coronavirus^4^.

The crystallographic structure of SARS-CoV-2 exhibits approximately 88% sequence identity with the other two coronaviruses found in bats (bat-SLCoVZC45 and bat-SL-CoVZXC21)^5^. For this reason, it is believed that the original host of the SARS-CoV-2 outbreak was bat-like^6^. Since discovery, an exponential growth in the number of cases of infections and deaths has been observed worldwide^3,7,8^. The World Health Organization (WHO) responded quickly to the COVID-19 threat by developing diagnostics and providing general guidance on patient monitoring, as well as up-to-date information; it also declared the outbreak a pandemic on March 11, 2020^8,9^.

The overall situation is progressing daily worldwide^10^. In order to further the development of prevention and control techniques, we must have a better comprehension of the nature of the pandemic^11,12^. It is important to know that SARS-CoV-2 replicates in the upper respiratory tract, and infected patients produce a multitude of virus particles which further contributes to the spread of infection^13^. Similar to MERS and SARS, there are no distinguishing clinical features of COVID-19, and symptoms overlap significantly with other severe acute respiratory infections^9,14–16^.

Clinical characterization protocols are now being collected on patients worldwide to better define the illness, in terms of its natural history, mode of transmission, clinical profiles, management, and specific risk factors, to prevent or overcome the damaging effects of the disease^9,17^. What is known so far is that a significant proportion of individuals infected by COVID-19 remain asymptomatic and are thus an unbeknownst potential source of infection^18,19^. In symptomatic patients, the characteristic symptoms of the disease usually start less than a week after infection, and consist of fever, cough, nasal congestion, and fatigue, along with other signs of an upper respiratory tract infection^19^.

**Figure 1:**
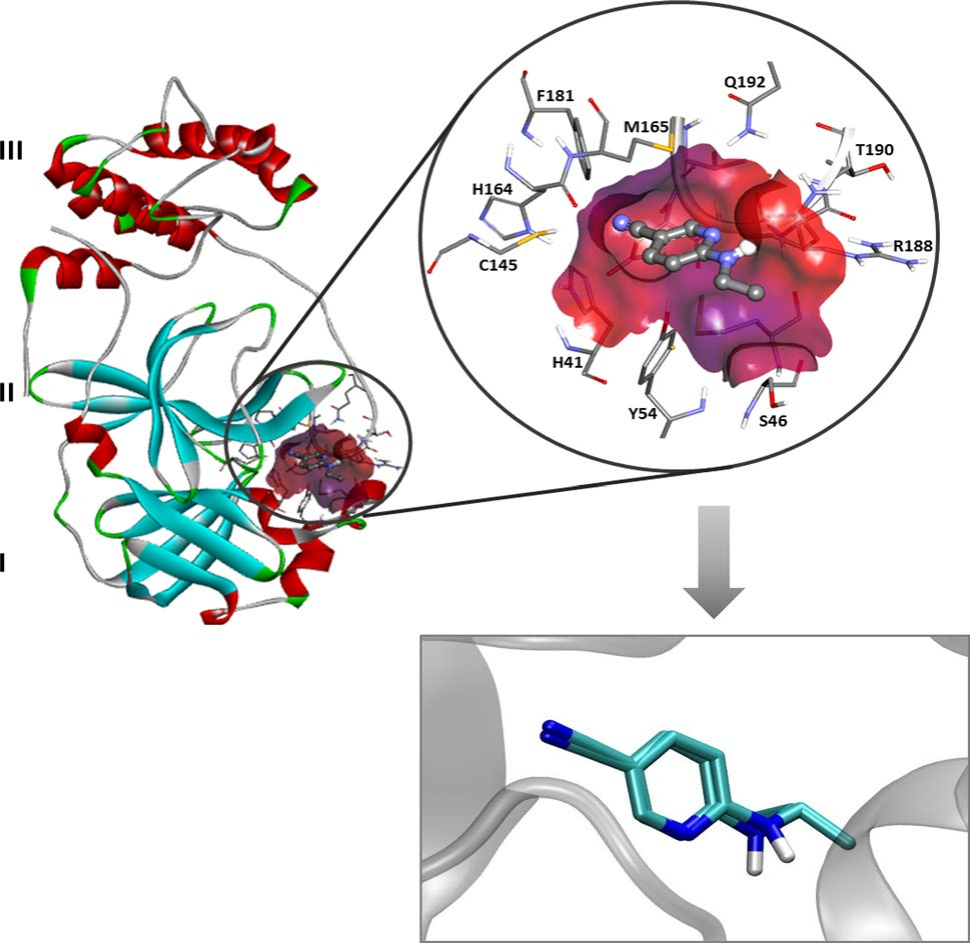
Crystallographic structure of the Covid-19 virus M^pro^ enzyme with the co-crystallized ligand 6-(ethylamino)pyridine-3-carbonitrile (PDB code 5R82) (up)^30^, and re-docking result of the co-crystallized ligand through AutoDock Vina^31^ as implemented in the MolAr software (down)^32^. Image generated in the Discovery Studio Software 4.5 https://discover.3ds.com/discovery-studio-visualizer-download.

In early 2003, SARS-CoV was revealed as the causative agent of the emergence of SARS^20,21^. The SARS virus's main proteinase (M^pro^), also known as SARS-CoV 3C-like protease (3CLpro), is a key enzyme responsible for the processing of viral polyproteins^21–23^. Together with the papain-like proteases, the M^pro^ is essential for the processing of polyproteins translated from the viral RNA^3,24^. In a structural analysis, the M^pro^ enzyme consists of three domains (Fig. 1). Domains I (residues 8–101) and II (residues 102–184) are well-known β-barrels, which together resemble the structure of chymotrypsin. Contrarily, domain III (residues 201–306) primarily consists of α-helices. Domains II and III, respectively, are connected by a long loop (residues 185–200). Also, located in a cleft between domains I and II, the M^pro^ active site presents a catalytic dyad formed by the conserved Cys145 and His41 amino acid residues. Equally important is the presence of a water molecule, which is a hydrogen atom bonded to His41; it can give rise to the third component of a catalytic triad^23^. It was indicated that domain III of M^pro^ is necessary for maintaining the proteolytic activity, which takes place by holding domain II and the long loop (residues 185–200) in a catalytically favorable orientation^25^ and/or orienting the N-terminal residues that play an important role for the catalytic activity of the enzyme^26^. To date, no human proteases with a similar cleavage specificity are known, suggesting that the inhibitors are unlikely to be toxic^3^. Based on this information, the present work has the main purpose of computationally designing new and more effective drugs to inhibition of the SARS-CoV-2 M^pro^^27–29^.

Researchers worldwide are undertaking the search for a vaccine while others search for a treatment regimen targeting SARS-CoV-2. Preliminary results demonstrated the application of both chloroquine (CQ) and hydroxychloroquine (HCQ) to be promising treatments for SARS-CoV-2^33–35^. CQ, for instance, exhibits inhibition of the SARS-CoV-2 infection at micromolar concentrations. Both compounds are classified as 4-aminoquinoline antimalarial drugs^36^. However, further studies are needed to ensure the administration of these medicines is safe. Given the exposure so far, this work aims to provide significant contributions to accelerate the discovery of novel and efficient remediation methods against the damaging effects of COVID-19.

Computational screening is now the prime focus for solving the crisis of SARS-CoV-2 infections. This is likely because such strategies reveal rational pathways for the development of fast and efficient drugs. In this regard, the combination of quantum mechanics and molecular mechanics calculations are robust tools for investigating a vast range of drug candidates, as well as identifying potential molecular targets for the sites of action of these therapeutic agents^37^. In this work, the nitro derivatives of quinoline (Q) and quinoline N-oxide (QO) were computationally investigated. The choice of these derivatives was predominantly based on the nitration reaction they undergo, which is characterized by the replacement of a hydrogen atom with a nitro group, and also because it is one of the most industrially used reactions, not requiring the use of sophisticated equipment to be performed^38–42^. This nitration reaction is therefore extremely attractive since synthesis requires low-cost materials and simple reaction paths, and any country can implement the large-scale manufacturing process for such products^38–42^. In this context, this research explores new therapeutic alternatives to combat the SARS-CoV-2 outbreak by utilizing computational simulations at the Density Functional Theory (DFT) level, molecular docking, and molecular dynamics methods as a well-reasoned strategy that provides insights on the physicochemical properties as well as the interaction and reactivity of these molecules as potential drug candidates.

## Results and discussion

**Figure 2:**
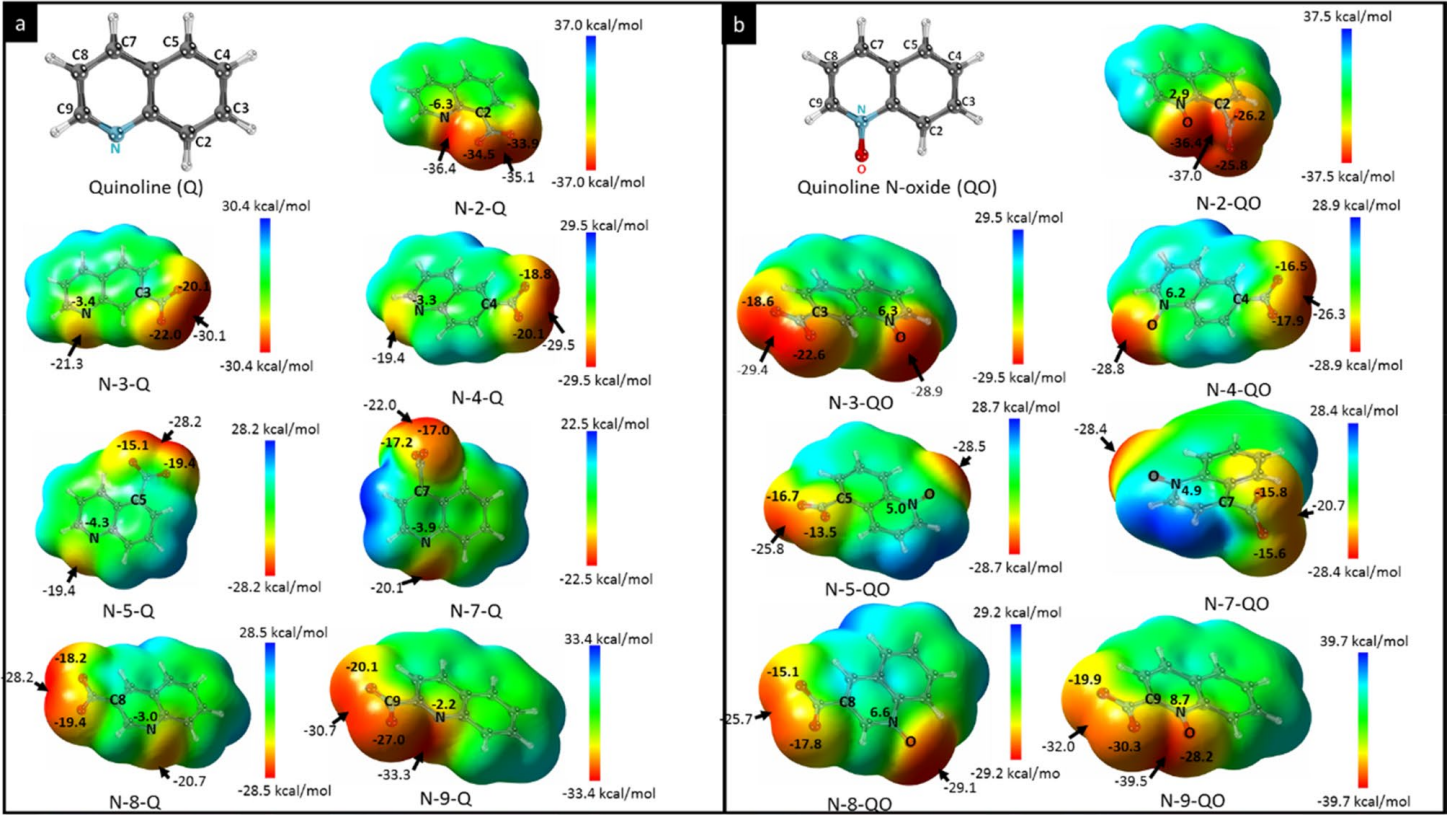
Optimised structures of nitro derivatives of (**a**) Q and (**b**) QO and computed electrostatic potential maps with contour value of 0.004. Image generated in the GaussView 6.0 https://gaussian.com/gaussview6/.

As a first step, we have performed DFT and TDDFT calculations for the nitro derivatives of compounds Q and QO to better understand their electronic structure, spectroscopic properties, and chemical reactivity. Figure 2 shows the electrostatic surface potential for the optimised structure of all the nitro derivatives of Q and QO, which have screened in this in silico study. Hence, we can also see that the charge distribution mainly depends on the various orientations of the nitro groups—regions with negative potential (red) that act as an excellent electron acceptor—that were added to the Q and QO compounds, respectively, with the specific objective of conferring the most favorable interaction between the drug and the target. Note also that the nitro group increases the polarity of these compounds, which is an attractive characteristic for pharmacological applications^43^. Additionally, nitro derivatives of QO compounds, in this case, have a more polarized structure. These slight structural changes are responsible for modulating the biological activity of these compounds, which may provide new clues for an in-depth interpretation of their microscopic behavior. These theoretical findings are consistent with the molecular docking simulations performed in this study.

All compounds were also identified in terms of computed IR-active modes and UV–Vis absorbance spectroscopy, as we show in Figure S1. These results can easily be used to distinguish the isomers obtained. In parallel, a comparison of the difference between the total electronic energy (ΔE) for the computed Q and QO isomers, presented in Table S1, suggests that both N-4-Q and N-4-QO compounds in terms of energy are more stable. Additionally, the HOMO–LUMO gaps reveal a minor difference of 4.07 to 4.31 eV for nitro derivatives of Q and of 3.11 to 3.69 eV for nitro derivatives of QO, respectively (see Table S1). In this case, a lower HOMO–LUMO gap value for QO derivatives, in principle, suggests greater reactivity for these isomers compared to Q derivatives. Figure S2 shows the shape of molecular orbitals (MOs) for all ligands studied. A detailed analysis of composition and localization of the MO reveals that the HOMO energies are, in principle, insufficient to describe the chemical behavior of these ligands. From the frontier effective-for-reaction molecular orbital (FERMO) concept, the reactions that are driven by HOMO, and those that are not, can be better explained for such compounds^44–47^. These findings are consistent with previous studies^46,47^.

To elucidate the modes through which our drug candidates interact SARS-CoV-2, the crystal structure of the M^pro^ of the virus in complex with 6-(ethylamino)pyridine-3-carbonitrile was downloaded for study^30^. Once the enzyme had been prepared, the molecular docking protocol was initiated. In the first part of this investigation, re-docking calculations were performed using the MolAr software^32^, with implementation of the AutoDock Vina program^31^. To determine the ideal docking parameters, these re-docking calculations were performed according to the orientation and conformation adopted by the experimental co-crystallised active ligand present in the binding pocket. It is important to notice that the M^pro^ enzyme used in this work was found in its native form.

The small RMSD variation (0.94) obtained from the re-docking calculations, suggested that the program was able to correctly and efficiently simulate the experimental results for the respective ligands. This preliminary outcome indicated that the conformational deviation of the molecular docking technique was suitable for our purposes and that the method was highly sensitive and specific. The re-docking overlap is presented in Fig. 1. To simulate the modes through which our drug candidates interact with the SARS-CoV-2 M^pro^ enzyme, we employed the best parameters provided by the data from the re-docking study carried out with the co-crystallised active ligand. All the computed interaction energy results are displayed in Table S2 and S3 in supplementary material.

As shown in Table S2, all the drug candidates studied (i.e., nitro derivatives of Q and QO) interacted well with M^pro^ active site, with interaction energy values in the range of − 4.3 to − 5.0 kcal mol^−1^. Some of the nitro-QO compounds, such as the inhibitors N-4-QO, N-9-QO, N-8-QO, together with QO, had slightly more stabilising interaction energy values than those of their corresponding nitro-quinolines (Table S2). In general, it is noteworthy that the compounds studied had a greater affinity for M^pro^ than the co-crystallised ligand did (the latter showing an interaction energy value of −3.9 kcal mol^−1^). In order to assess the potential of such findings, using the same protocol, docking procedures were performed with the commercial drugs CQ and HCQ, which are currently adopted for the treatment of SARS-CoV-2 infection, and their interaction energy values were found to be − 2.8 and − 2.3 kcal mol^−1^, respectively. A remarkable trend could be observed from these outcomes. Note that all of our drug candidates presented lower interaction energy values than CQ and HCQ, with a significant energy difference, of up to 2 kcal mol^−1^. Additionally, our study showed that the many of the nitro-QO compounds led to a more stabilising interaction energy in the M^pro^ active site. Based on these findings, we also investigated the chloroquine and hydroxychloroquine N-oxides forms (denoted as CQO and HCQO), which displayed a significant improvement in interaction energy values of − 3.0 and − 3.1 kcal mol^−1^, respectively. Interestingly, the interaction energy of HCQO was almost 1 kcal mol^−1^ more stabilizing than that of HCQ. This trend was deeply analyzed using molecular dynamics (MD) techniques. The influence of the N-oxide group was also investigated at different sites and through different combinations for the CQO and HCQO compounds (Table S3). According to that table, with all combinations investigated, we can observe that no improvement in interaction energy was detected for CQO. On the other hand, for HCQO, the presence of the N-oxide group at some sites led to slightly more favorable interaction energies. See table S3 for more details. Herein, our main goal was to determine whether the inhibitors studied could target the M^pro^ enzyme. The molecular docking pose of each drug candidate indicated that they could indeed fit accurately within the substrate-binding pocket.

**Figure 3:**
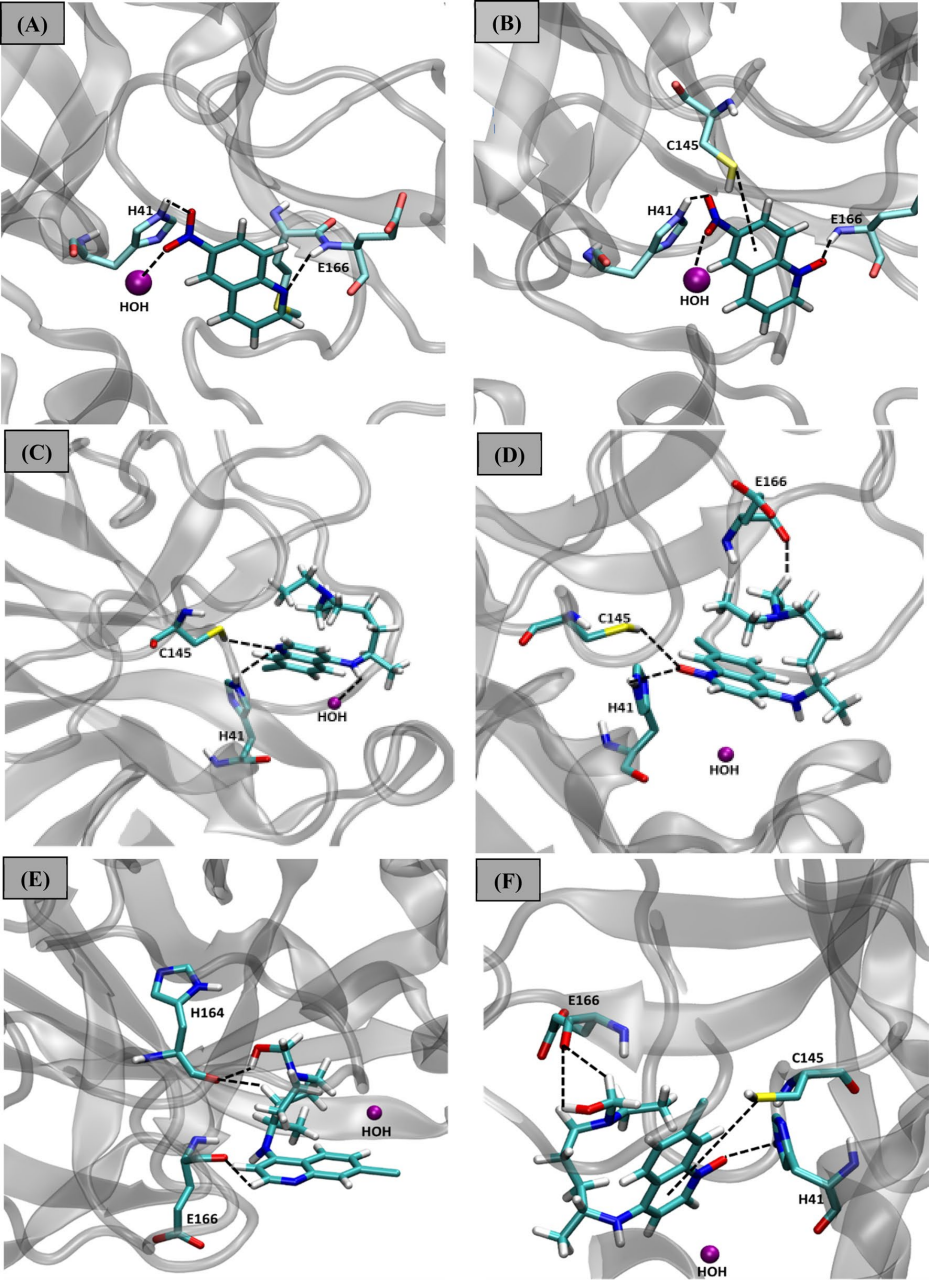
Intermolecular interactions performed by the inhibitors (**A**) N-4-Q, (**B**) N-4-QO, (**C**) CQ, (**D**) CQO, (**E**) HCQ and (**F**) HCQO in the M^pro^ active site. Image generated in the Visual Molecular Dynamics 1.9.3 https://www.ks.uiuc.edu/Research/vmd/.

In the case of SARS-CoV-2 virus M^pro^ enzyme, the protomer is composed of three domains, as commented previously (see Fig. 1). The enzyme has a Cys145–His45 catalytic dyad, and the substrate-binding pocket is known to be located in a cleft between domains I and II^48^. Hence, the structural features determined from these data are important for guiding our assessment of the interaction modes of the inhibitors in the M^pro^ active site. As shown in Fig. 3, the N-4-QO performed hydrogen bonds with all the residues and the water molecule of the catalytic triad. In fact, these specific interactions constitute one of the parameters analysed in this docking study. This same trend is not observed for inhibitor N-4-Q, suggesting that the N-oxide version of this ligand adopts a more favourable conformation which allows for its interaction with the catalytic triad, resulting in a slightly more stabilizing interaction energy. Similarly, the interactions performed by the other ligands can also be observed in Fig. 3.

From the molecular docking calculations, it was possible to deduce that our drug candidates had a more stabilizing interaction energy effect than CQ and HCQ in the M^pro^ binding pocket. To better assess the interaction modes of our inhibitors, N-4-Q and N-4-QO were chosen as representatives of the set for MD simulations. Likewise, the same calculations were performed for CQ and HCQ and their N-oxides CQO and HCQO (see Figure S3).

Additionally, in this study, the dynamic behavior of complexes M^pro^/N-4-Q, M^pro^/N-4-QO, M^pro^/CQ, M^pro^/CQO, M^pro^/HCQ, M^pro^/HCQO inside the SARS-CoV-2 M^pro^ enzyme was investigated. The extracted frame, which was considered the representative conformational structure for all inhibitors throughout the MD simulation, corresponds to the average of the RMSD value. By analyzing the results of the RMSD plots, it was observed that most of the deviations from the N-4-Q and N-4-QO structures were very small, not exceeding 0.5 Å, i.e., these ligands are well-accommodated in the SARS-CoV-2 M^pro^ active site according to Figures S4 and S5.

**Figure 4:**
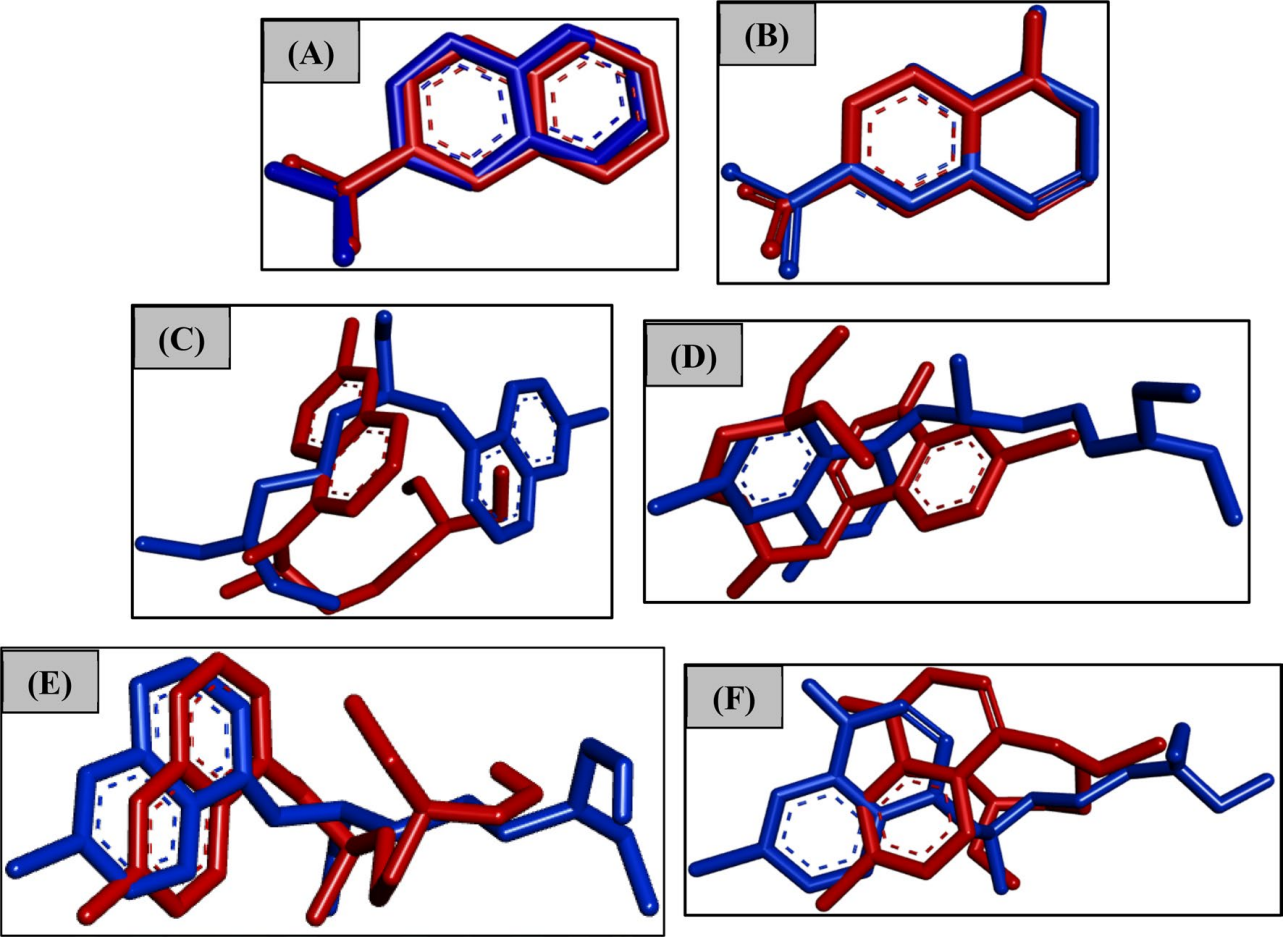
Overlap of the initial (red) and representative (blue) structures of the 20-ns simulation of MD. Image generated in the Discovery Studio Software 4.5 https://discover.3ds.com/discovery-studio-visualizer-download.

To get more insights into the intrinsic reactivity of each one of these ligands, in this study, we have performed the analysis of the strain effect along the MD simulation. Since these factors have a pivotal role and affect the reactivity of these ligands^49,50^. In the present study, the strain effect along the MD simulation can be clearly visualized by the overlap of the initial (red) and representative (blue) structures obtained after 20 ns of simulation, as shown in Fig. 4. Based on that figure, we notice that our compounds N-4-Q and N-4-QO showed a slight bending at the quinoline ring (strain), which makes this compound in principle more reactive, resulting in a small oscillation according to the RMSD graphs (Figures S4 and S5). Importantly, this trend is essential because it indicates a low variation of strain (deformation of the ligand along simulation), reaching a stabilizing conformation more quickly. On the other hand, due to larger molecular mass and bulk of the CQ, HCQ and their corresponding oxides, there was a very higher variation of strain (Fig. 4C–F). We believe that this slight strain can induce a high intrinsic reactivity for these ligands.

**Figure 5:**
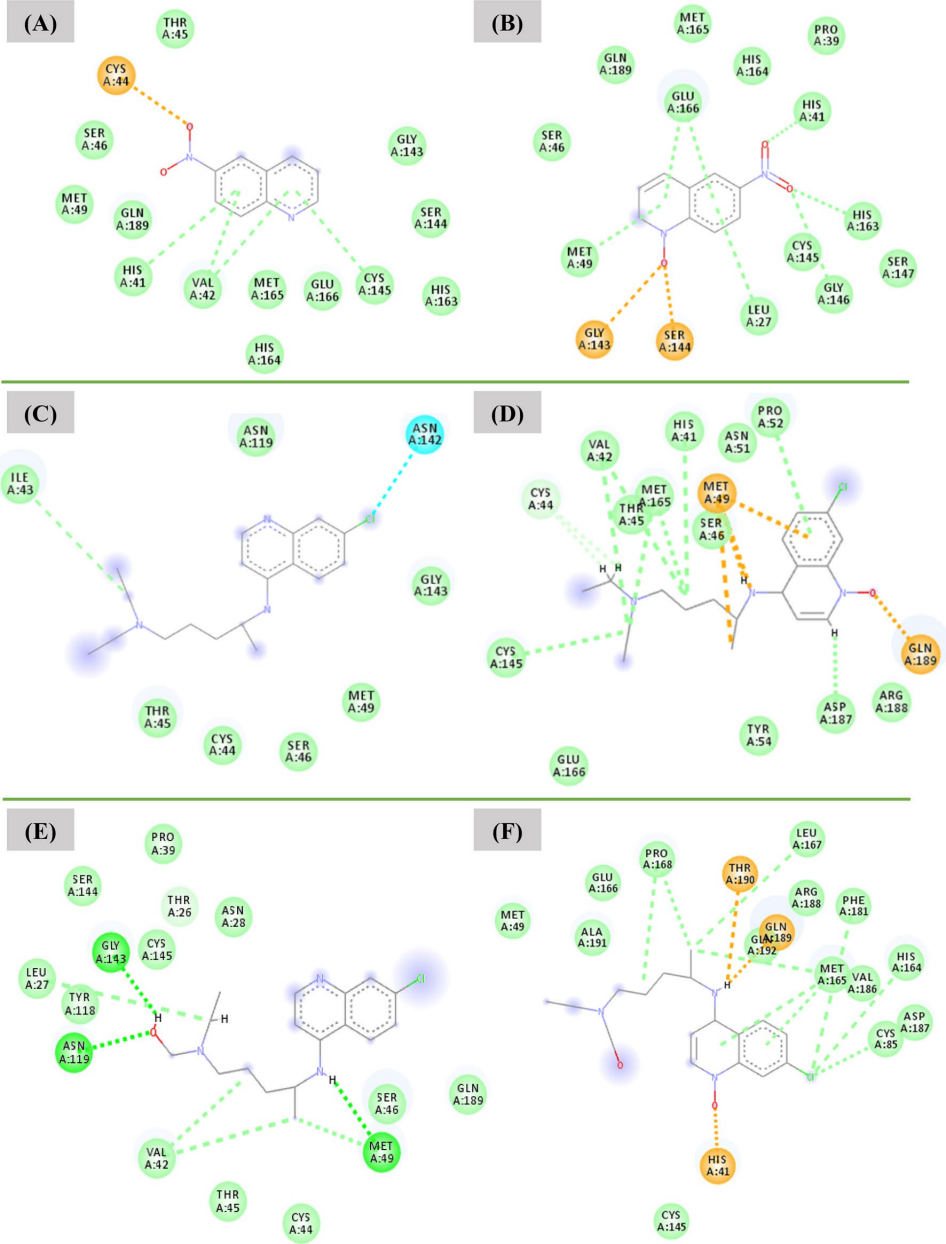
Interactions performed during 20 ns in the MD simulation with the (**A**) M^pro^/N-4-Q, (**B**) M^pro^/N-4-QO, (**C**) M^pro^/CQ, (**D**) M^pro^/CQO, (**E**) M^pro^/HCQ, (**F**) M^pro^/HCQO complexes. Image generated in the Discovery Studio Software 4.5 https://discover.3ds.com/discovery-studio-visualizer-download.

As shown in Fig. 5A, the N-4-Q compound performed hydrogen bond interaction with Cys44 (2.78 Å) and hydrophobic interactions with Thr45, Ser46, Met49, Gln189, His41, Val42, Met165, Glu166, His164, Cys145, His163, Ser144, Gly143, respectively. These interactions are essential in inhibiting the enzymatic activity of M^pro^ and are in accordance with some other studies^51–53^, as well as the docking results of this work. By analyzing the graph of hydrogen interactions (see Figure S4), we found that the compound N-4-Q performed up to three hydrogen bond type interactions. However, there was only one effective interaction that occurred during the entire 20 ns of MD simulation, which is according to the pharmacophoric map (see Fig. 5A). In turn, the N-4-QO compound was stabilized by four hydrogen bonds with His41 (2.93 Å), His163 (2.72 Å), Gly143 (3.01 Å), Ser144 (3.04 Å), and hydrophobic interactions with Met49, Ser46, Gln189, Glu166, Met165, His164, Pro39, Leu27, Cys145, Gly146, and Ser147, respectively, as shown in Fig. 5B. According to Zhang and coworkers^53^, in the catalytic site, the residues Glu166, His41, and Gys145, respectively, are key species of the target protease. Thus, the interaction of these amino acids with inhibitors is essential for blocking the enzymatic activity of M^pro^. Additionally, it is observed that the N-4-QO can make up to three bonds during the trajectory; however, occurs only one hydrogen bond in most of the entire simulation (Figure S5).

In the case of the dynamic behavior of both CQ and CQO compounds, we have observed that CQ remained unstable over the 20 ns of simulation, as shown in Figures S6. On the other hand, CQO remained stable during the same period of simulation. The CQ compound presented large oscillations in the M^pro^ active site, by rotating the N-diethyl-pentane-1,4-diamine portion. This ligand has many rotatable bonds, and because of the exposure to the solvent, this increases the ligand flexibility, making it more unstable in the active site. For the simulation with CQO, this ligand was better accommodated in the M^pro^ site over the 20 ns of simulation, and this fact leads us to believe that this oxo form significantly contributes to the compound stabilization. Although the RMSD deviation was high when compared to N-4-Q and N-4-QO compounds, they are coherent since the chemical structures of CQ and CQO are bulkier and had a larger molecular mass, as well as several rotatable bonds. Consequently, there is a change in the conformation (Fig. 4C,D), further increasing the flexibility of the inhibitors, and therefore is expected a more significant oscillation in the RMSD (see Figures S6 and S7). Through the pharmacophoric map, as shown in Fig. 5C, hydrophobic interactions can be observed with the residues Asn119, Ile43, Thr45, Cys44, Ser46, Met49 and Gly143. We can also notice a halogen bond with the Asn142 amino acid residue (3.22 Å). On the other hand, in the case of CQO, this inhibitor performed hydrogen bonds with Gln189 (2.78 Å) and Met49 (2.30 Å), together with several hydrophobic interactions, specifically with the residues Glu166, Cys145, Cys44, Val42, Thr45, Met165, His41, Ser46, Met49, Asn51, Pro52, Tyr54, Asp187 and Arg188 (see Fig. 5D). These results are in accordance with the Hydrogen bond graph, since up to two hydrogen bonds are observed during the 20 ns of simulation (Figure S7).

Likewise, the HCQ compound remained stable in the M^pro^ active site after 7.0 ns of simulation, mainly due to many conformational changes (Figure S8), such as in relation to the amino-pentyl(ethyl)aminoethanol group that underwent rotation, resulting in a more energetically favourable conformation compared to its initial chemical structure, i.e., thus decreasing the RMSD value (Figure S8 and Fig. 5E). However, the final configuration of HCQ had less strain than CQ. Therefore, we can speculate that the minimum strain, which is associated with a low RMSD value, is directly related to the toxicity of these compounds.

As shown in Fig. 5E, the HCQ exhibit one hydrogen bond with Gly143 (2.10 Å), Asn119 (2.46 Å) and Met49 (2.30 Å), and several hydrophobic interactions with the residues Tyr118, Leu27, Ser144, Cys145, Pro39, Thr26, Asn28, Val42, Thr45, Cys44, Ser46 and Gln189, respectively. Through the Hydrogen bond graph, it was observed that for this compound after equilibration in the active site, up to three hydrogen bonds could be accomplished (Figure S8). While the HCQO compound showed the RMSD value of around 2.5 Å, due to the structural distortions in the N-diethyl-pentane portion during the 20 ns of simulation (Figure S9). The oscillation of the ligand in the site resulted in a less energetically favorable conformation compared to its initial chemical structure (Fig. 4F). Also, this compound performed three hydrogen bond interactions with Thr190 (2.98 Å), Gln189 (1.89 Å) and His41 (1.70 Å) and hydrophobic interactions with Cys145, Met49, Ala191, Glu166, Pro168, Leu167, Arg188, Gln192, Phe181, Met165, Val186, His164, Cys85 and Asp187, respectively, as we show in Fig. 5F. Considering the hydrogen graph (Figure S9), the HCQO can make up to five hydrogen bonds.

In order to confirm the structural stabilization in the simulation environment, the RMSF was calculated from the average position of each amino acid residue of M^pro^ (Figures S4–S9). Higher RMSF values indicate that the residues have undergone major changes, corresponding to regions of loops. On the other hand, for the residues of the active site region and the alpha-helices/beta sheets regions, there is a lower RMSD value, thereby revealing the increased stability of these areas. The regions of loop are freely exposed to the solvent to a larger degree, and according to the graphs, we can observe that the sidechain has the largest variation of RMSF, indicating greater degree of freedom, that is, larger flexibility. In addition, the backbone presented a low variation of RMSF, this is expected because these residues are found in central regions of the protein, for example, inside the active cavity. Finally, it was possible to notice, from the RMSF values of the protein structure, the inexistence of large oscillations, maintaining itself conserved during the whole process of simulation.

From the MD simulation, we have estimated the interaction energies for all cases studied: N-4-Q (− 96.54 kJ mol^−1^), N-4-QO (− 107.35 kJ mol^−1^), CQ (− 100.65 kJ mol^−1^), CQO (− 82.27 kJ mol^−1^), HCQ (− 116.60 kJ mol^−1^), HCQO (− 148.20 kJ mol^−1^). An important outcome observed in this study is that the majority of the N-oxide compounds had an energetically more favorable affinity at the M^pro^ active site than their Q counterparts. These findings are consistent with the molecular docking calculations. The existence of intermolecular interactions strongly guides these trends. Note that the N-4-QO performed more hydrogen bonds than N-4-Q, as shown in Fig. 5. This fact helps explain the more stabilizing interaction energy found for N-4-QO. From the pharmacophoric maps shown in Fig. 5, the accomplishment of hydrogen bonds, along with the hydrophobic interactions, are key to understand the biological activity of these inhibitors.

Yet, the success of a novel drug candidate is commonly attributed to diverse factors, including their bioactivity, rich pharmacokinetic (PK) and pharmacodynamics (PD) profiles, as well as toxicity. It would be therefore of huge interest to investigate these properties in the preliminary stages to in silico design of safer and more efficient drugs. Hence, the ADMET evaluations involve sequential and iterative assessments of the efficacy, PK, PD, metabolic and toxicological properties in the model of potential drug candidates^54^. From the ADMET results, the theoretical parameters of toxicity (LD_50_) were obtained for each compound: N-4-Q (2.53 mol kg^−1^), N-4-QO (2.56 mol kg^−1^), CQ (2.95 mol kg^−1^), CQO (2.68 mol kg^−1^), HCQ (2.66 mol kg^−1^), HCQO (2.69 mol kg^−1^). We can observe that the parameter toxicity slightly varied from N-4-Q to N-4-QO, suggesting that the toxicity of these compounds is essentially equal. Similarly, this trend also is observed for HCQ and its corresponding N-oxide (HCQO). On the other hand, we have noticed a more significant variation for CQ and CQO compounds, indicating that CQO theoretically presents a higher level of toxicity. In addition, these molecular calculations also showed that HCQ is more toxic than CQ. Yet, this trend does not corroborate with previous experimental results^55^. It is essential to highlight that the molecular results obtained do not take into account the effects of the counterion and, for this reason, suggest a different trend to the experimental findings previously reported^55^. In particular, this divergence most-likely is related to the fact that the commercially used HCQ is a salt-based on hydroxychloroquine sulfate, while the CQ used is a salt-based on chloroquine diphosphate. It additionally is well-known that the counterion has a substantial effect not only on its biological activity but also on the toxicity of such compounds as well^56–59^. Therefore, our molecular results indicate that the presence of phosphate groups contributes to increasing the toxicity of CQ in the treatment of the SARS-CoV-2 infection. Consequently, we can conclude that for the same type of salt used, it is expected CQ to be less toxic than HCQ, according to the molecular trend observed in this study.

In the last part of this investigation, we carried out new molecular docking calculations with three selected α-ketoamide derivatives (known inhibitors of coronavirus protease enzymes)^60^. Consequently, this strategy might provide a more detailed data comparing their interaction modes in the M^pro^ active site for these drugs designed. As such, the chemical structures of the α-ketoamide inhibitors and biological activities are shown in Figure S10 of supplementary material. Based on the newly obtained results, the compounds 11n, 11r and 11 s exhibited interaction energies of approximately − 6.4 kcal mol^−1^, − 6.9 kcal mol^−1^ and − 7.0 kcal mol^−1^, respectively. From these results, note that these compounds showed slightly more stabilized interaction energies in comparison with those of our drug candidates. As such, the intermolecular interactions with residues from the active site can be observed with more details in Figure S11. In parallel, from the ADMET analysis can be observed that α-ketoamide compounds showed LD_50_ values of 2.56 mol kg^−1^ (for 11n), 2.33 mol kg^−1^ (for 11r) and 2.43 mol kg^−1^ (for 11s). These results suggest that α-ketoamide in comparison to our compounds is likely more toxic. In face with these theoretical outcomes, we can notice that our drug candidates demonstrate potential to be used as therapeutic agents for the COVID-19 treatment.

## Conclusion

We conclude that this in silico study to contribute toward the rational design of new and more efficient drugs for the treatment of SARS-CoV-2 infection. Hence, the most important lesson from this structure-based study was that the QO derivatives are better inhibitors than their Q counterparts. In light of these results, we can suggest that in vitro and in vivo experiments be urgently carried out to investigate the nitro derivatives of QO further, since there is as yet no efficient treatment for this disease. Finally, we emphasise that these compounds can be easily produced on a large scale (at a low-cost), making them a promising treatment option against SARS-CoV-2 infection.

## Methods

### Datasets

Herein, the crystal structure dataset for SARS-CoV-2 virus M^pro^ enzyme was obtained from the Protein Data Bank (PDB; accession ID: 5R82, resolution 1.31 Å)^30^. Then, full optimizations and frequencies of nitro derivatives of Q and QO were achieved at B3LYP level of theory with 6-31+g(d,p) basis set in the Gaussian 09 package^61^. For a better description of the electronic parameters, it was also performed single-point energy Time-Dependent DFT (TD-DFT) calculations at B3LYP/6-31+g(d,p) level.

### Molecular docking

The molecular docking was conducted with the tool AutoDock Vina (version 1.1.2)^31^, as implemented in the MolAr (Molecular Architecture) software^32^. For the crystallographic M^pro^ structure preparation, the loop regions were rebuilt using the Modeller^62^. The ions and water molecules were removed from the original PDB, with the exception of water molecules that were in the M^pro^ active site. The addition of polar hydrogen atoms were performed according to the protonation state of the receptor at pH 7.4, by using the Chimera software^63^. For the docking protocol, the M^pro^ enzyme and the structures of Q and QO derivatives were used as receptor and ligands, respectively. The grid box was centered on the co-crystallized ligand (6-[ethylamino]pyridine-3-carbonitrile) of SARS-CoV-2 virus M^pro^ enzyme (5R82), and the coordinates were x = 12.053, y = − 0.871 and z = 24.157, with 1 Å spacing. Docked poses were then selected on the basis of scoring functions and protein − ligand interactions. Binding interaction figures were generated using Discovery Studio 2017 R2^64^. AutoDock Vina employs the Iterated Local Search global optimizer^31^.

### Molecular dynamics simulations

In a further theoretical insight, the key docking complexes were evaluated by molecular dynamics (MD) simulation using the GROMOS54A7 all-atom force field^65^ and performed using GROMACS 5.1 software^65,66^. The M^pro^ complexes were inserted into a 12 Å water box with the SPC solvation model, and sodium and chlorine ions were added for charges neutralization under periodic boundary conditions. The calculation of electrostatic interactions was then performed by using the Particle Mesh Ewald method with a cut-off of 12 Å and time step of 1 fs. Initially, complexes were minimized over 5000 cycles using the steepest descent algorithm. After the minimization, a 500 ps equilibration was done in the NVT ensemble slowly increasing the temperature from 50 to 300 K, using Berendsen thermostat. In order to equilibrate the pressure of the system, a NPT equilibration was performed employing Parrinello–Rahman barostat^67^ to maintain the system pressure of 1 bar. After the equilibration of the systems, they were submitted to a MD production step with 20 ns of simulation and a 1 fs integration time. Atom trajectories were analyzed using Visual Molecular Dynamic (VMD, version 1.9.3)^68^. Due to the experimental inexistence of acute toxicity data for these compounds, in principle, we also provide a theoretical estimation for the LD_50_ values from the using of a rat model-based admetSAR predictor, which is freely available online at http://lmmd.ecust.edu.cn:8000/.

## Supplementary information

**Supplementary information.**
