# Supplementary material for: Computational evidence for nitro derivatives of quinoline and quinoline N-oxide as low-cost alternative for the treatment of SARS-CoV-2 infection
Source: Sci Rep. 2021 Mar 18;11:6397. doi: 10.1038/s41598-021-85280-9 (PMC7973710; doi:10.1038/s41598-021-85280-9)
Supplement: Supplementary file 1 — Supplementary information. [file 41598_2021_85280_MOESM1_ESM.docx]

**SUPPLEMENTARY MATERIAL**

**Computational evidence for nitro derivatives of quinoline and quinoline N-oxide as low-cost alternative for the treatment of SARS-CoV-2 infection**

Letícia C. Assis,^1^ Alexandre A. de Castro,^1^ João P. A. de Jesus,^2^ Eugenie Nepovimova,^3^ Kamil Kuca,^3,*^ Teodorico C. Ramalho^1,3^ and Felipe A. La Porta^2,*^

^1^ Department of Chemistry, Federal University of Lavras, CEP 37200-000 Lavras, Minas Gerais, Brazil

^2^ Laboratório de Nanotecnologia e Química Computacional, Universidade Tecnológica Federal do Paraná, 86036-370, Londrina, PR, Brazil;

^3^ Department of Chemistry, Faculty of Science, University of Hradec Kralove, Rokitanskeho 62, 500 03 Hradec Kralove, Czech Republic

^*^ Lead Contact: felipelaporta@utfpr.edu.br or [felipe_laporta@yahoo.com.br](mailto:felipe_laporta@yahoo.com.br)

kamil.kuca@uhk.cz

| System | ΔE (kcal/mol) | Bandgap  E_g_ (eV) | Hardness  η (eV) | Softness  S (eV) | Electronegativity  x (eV) | Electrophilicity  ω (eV) |
| --- | --- | --- | --- | --- | --- | --- |
| N-2-Q | 9,65 | 4.15 | 2.08 | 0.48 | -5.07 | 51.36 |
| N-3-Q | 0,52 | 4.24 | 2.12 | 0.47 | -5.22 | 54.47 |
| N-4-Q | 0,00 | 4.23 | 2.11 | 0.47 | -5.23 | 54.73 |
| N-5-Q | 3,64 | 4.20 | 2.10 | 0.48 | -5.24 | 54.98 |
| N-7-Q | 7,76 | 4.31 | 2.15 | 0.46 | -4.99 | 49.73 |
| N-8-Q | 1,32 | 4.07 | 2.04 | 0.49 | -5.25 | 55.05 |
| N-9-Q | 1,51 | 4.19 | 2.09 | 0.48 | -5.15 | 52.96 |
| System | ΔE (kcal/mol) | Bandgap  E_g_ (eV) | Hardness  η (eV) | Softness  S (eV) | Electronegativity  x (eV) | Electrophilicity  ω (eV) |
| N-2-QO | 11.75 | 3.69 | 1.84 | 0.54 | -4.53 | 41.04 |
| N-3-QO | 0.15 | 3.24 | 1.62 | 0.62 | -4.93 | 48.59 |
| N-4-QO | 0.00 | 3.18 | 1.59 | 0.63 | -4.92 | 48.46 |
| N-5-QO | 3.63 | 3.11 | 1.56 | 0.64 | -4.93 | 48.66 |
| N-7-QO | 7.56 | 3.56 | 1.78 | 0.56 | -4.72 | 44.65 |
| N-8-QO | 2.14 | 3.19 | 1.60 | 0.63 | -5.00 | 50.00 |
| N-9-QO | 12.08 | 3.49 | 1.75 | 0.57 | -5.02 | 50.48 |

**Table S1.** Analysis of electronic properties of nitro derivatives of Q and QO.

| **** | | | |
| --- | --- | --- | --- |
| **Nitro group position** | **Interaction Energy**  **(kcal mol^-1^)** | **Nitro group position** | **Interaction Energy (kcal mol^-1^)** |
| N-4-QO | -5.0 | N-4-Q | -4.6 |
| N-9-QO | -5.0 | N-9-Q | -4.6 |
| N-7-QO | -4.9 | N-7-Q | -5.0 |
| N-2-QO | -4.8 | N-2-Q | -4.8 |
| N-5-QO | -4.8 | N-5-Q | -4.8 |
| N-8-QO | -4.8 | N-8-Q | -4.6 |
| N-3-QO | -4.5 | N-3-Q | -4.5 |
| QO  CQO  HCQO | -4.5  -3.0  -3.1 | N-1-Q  CQ  HCQ | -4.3  -2.8  -2.3 |

**Table S2.** Interaction energy (in kcal mol^-1^) of nitro derivatives computed through AutoDock Vina program.

*Q = quinoline, QO = quinoline N-oxide, CQO = chloroquine N-oxide, CQ = chloroquine, HCQO = hydroxychloroquine N-oxide, HCQ = hydroxychloroquine, N = nitro group.

**Table S3.** Interaction energy (in kcal mol^-1^) of chloroquine and hydroxychloroquine derivatives computed through AutoDock Vina program.

|  | | | |
| --- | --- | --- | --- |
| **Oxide group position** | **Interaction Energy**  **(kcal mol^-1^)** | **Oxide group position** | **Interaction Energy (kcal mol^-1^)** |
| Sites 1,2 | -2.9 | Sites 1,2 | -3.2 |
| Sites 1,3 | -2.4 | Sites 1,3 | -2.9 |
| Sites 1,2,3 | -2.6 | Sites 1,2,3 | -2.8 |
| Sites 2 | -3.0 | Sites 2 | -3.4 |
| Sites 2,3 | -3.0 | Sites 2,3 | -3.3 |
| Sites 3 | -2.9 | Sites 3 | -3.3 |
|  |  |  |  |


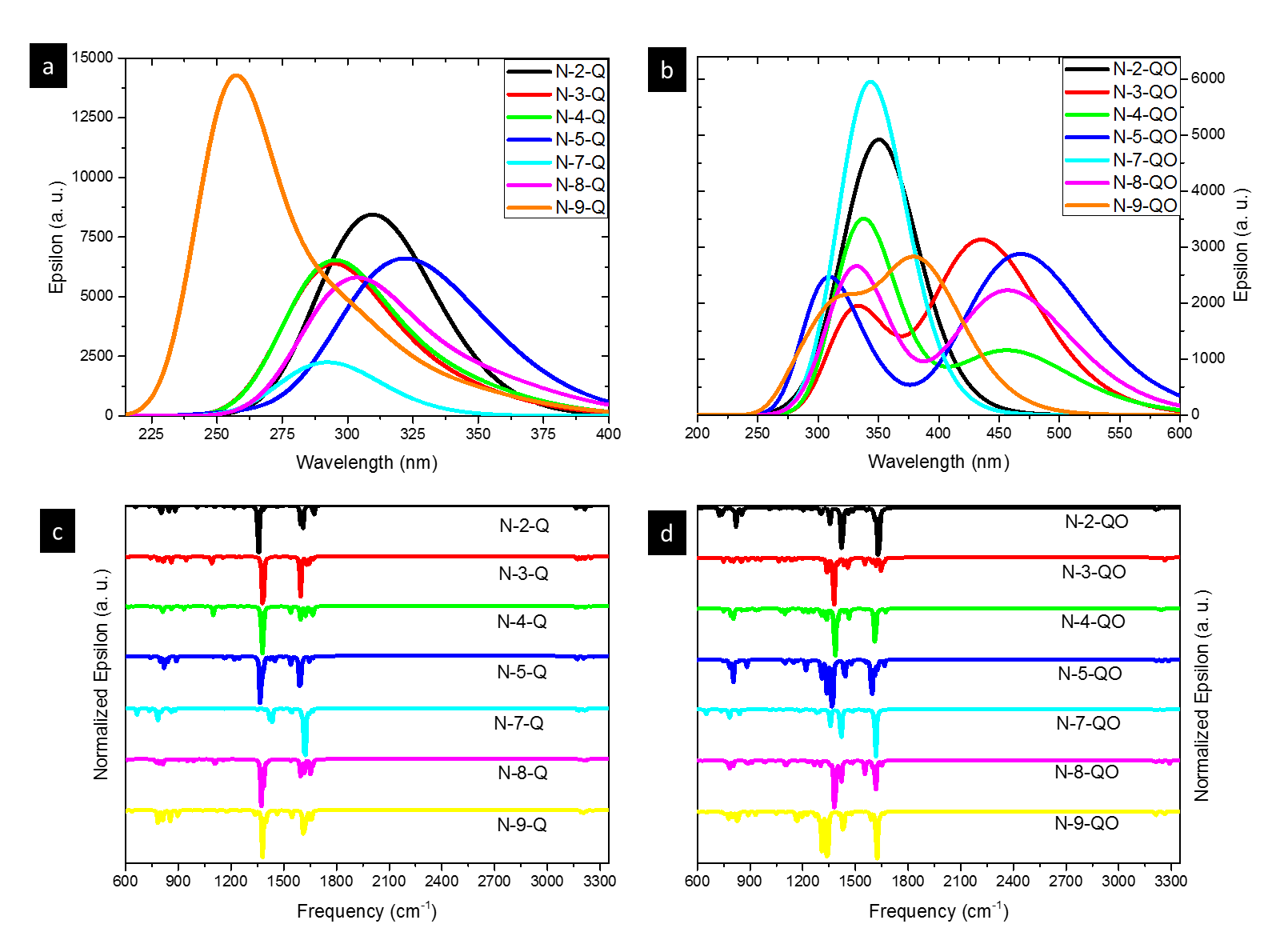


**Figure S1**. Simulated (a and b) UV-vis and (c and d) IR spectra of nitro derivatives of Q and QO.


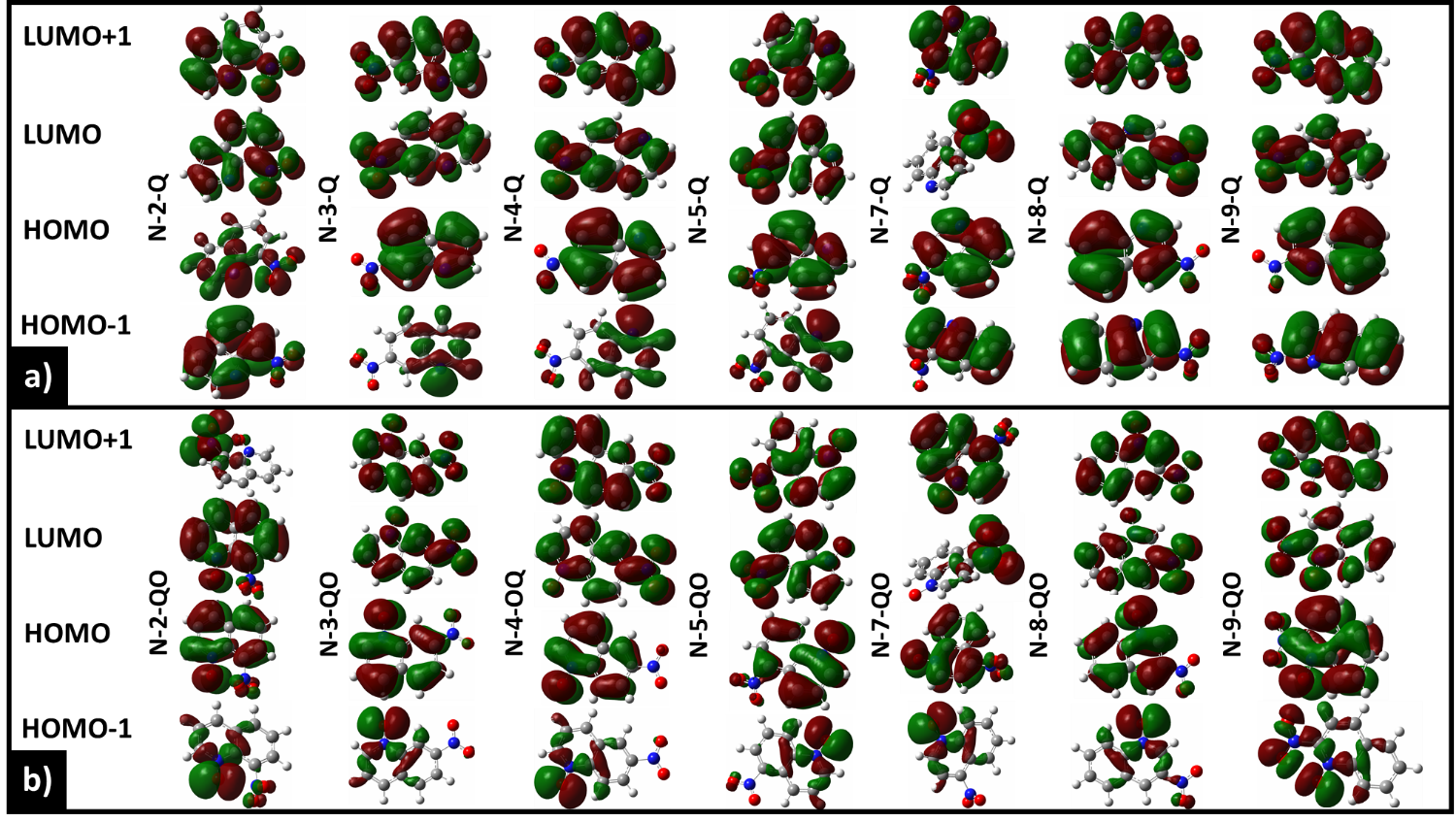


**Figure S2**. Frontier molecular orbital representation (with a contour value of 0.020) for the nitro derivatives of Q and QO.





**Figure S3.** Chemical structures of the species employed in the MD simulations.


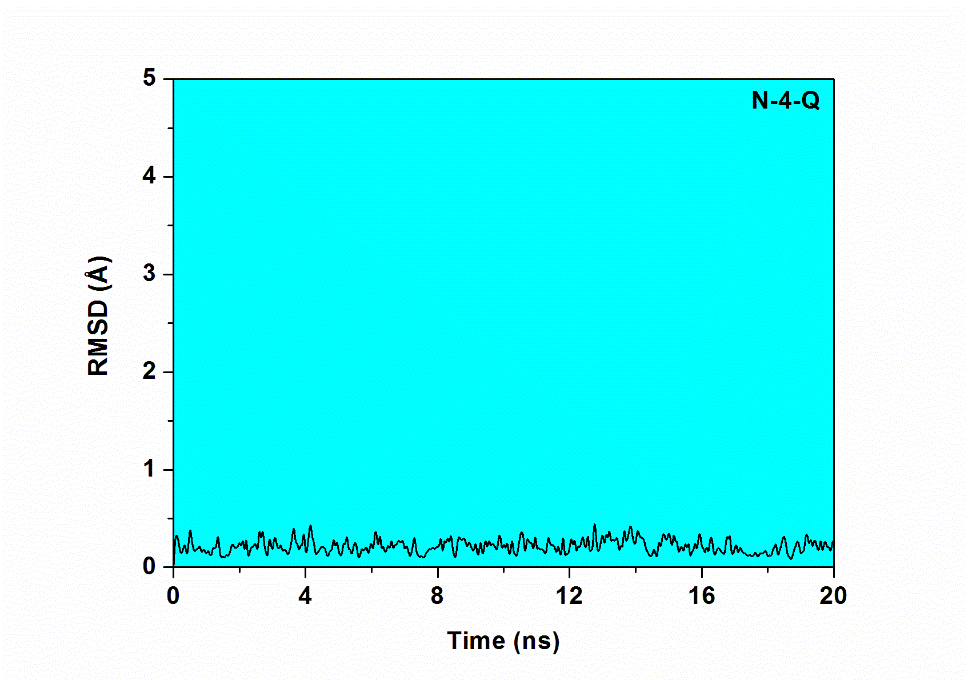

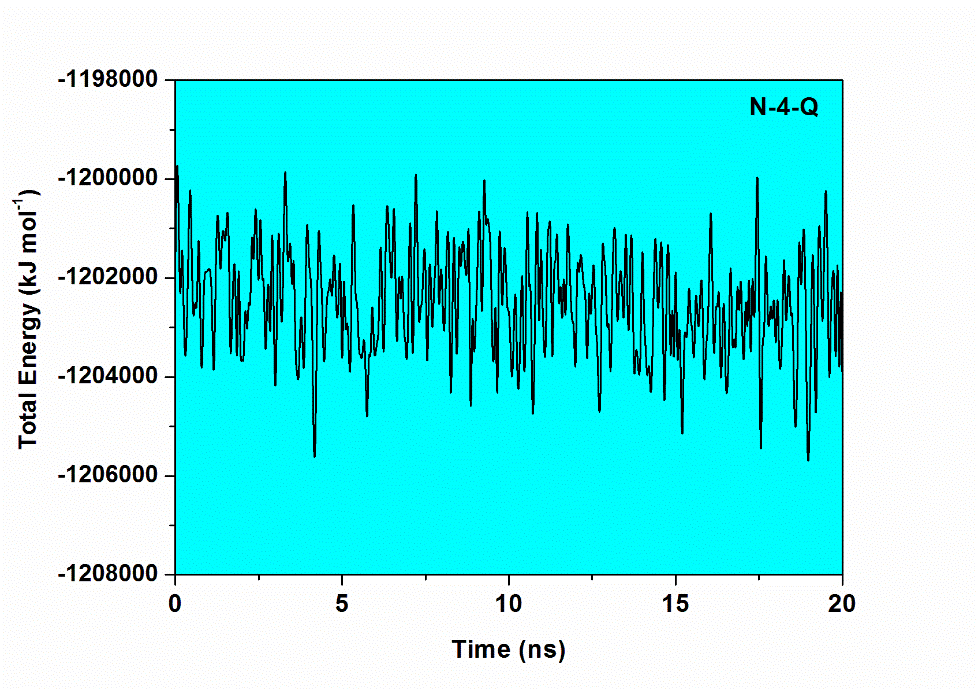

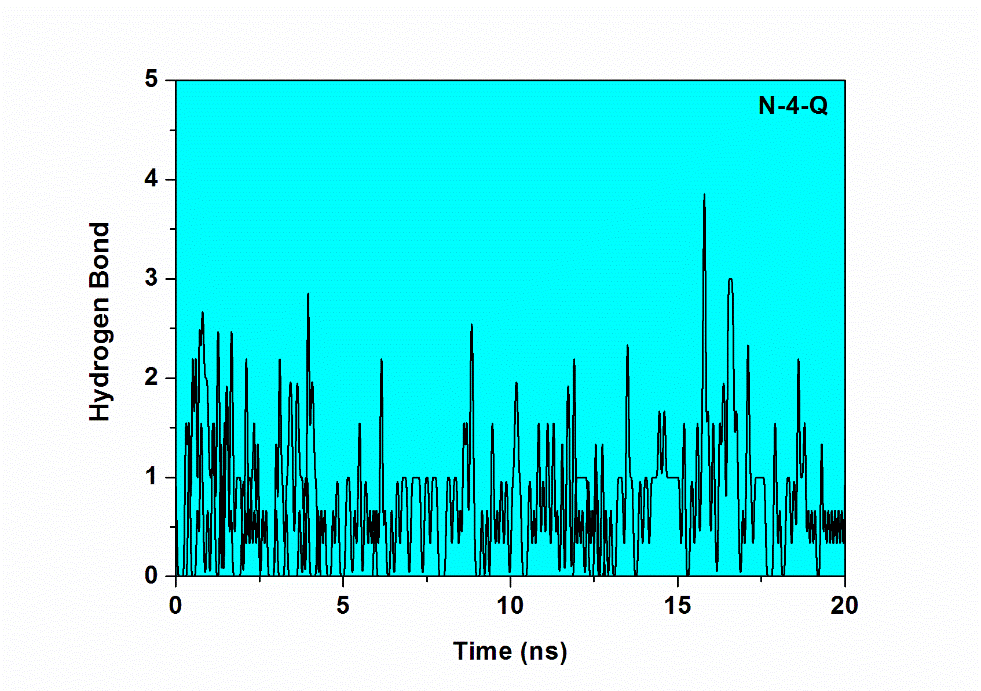

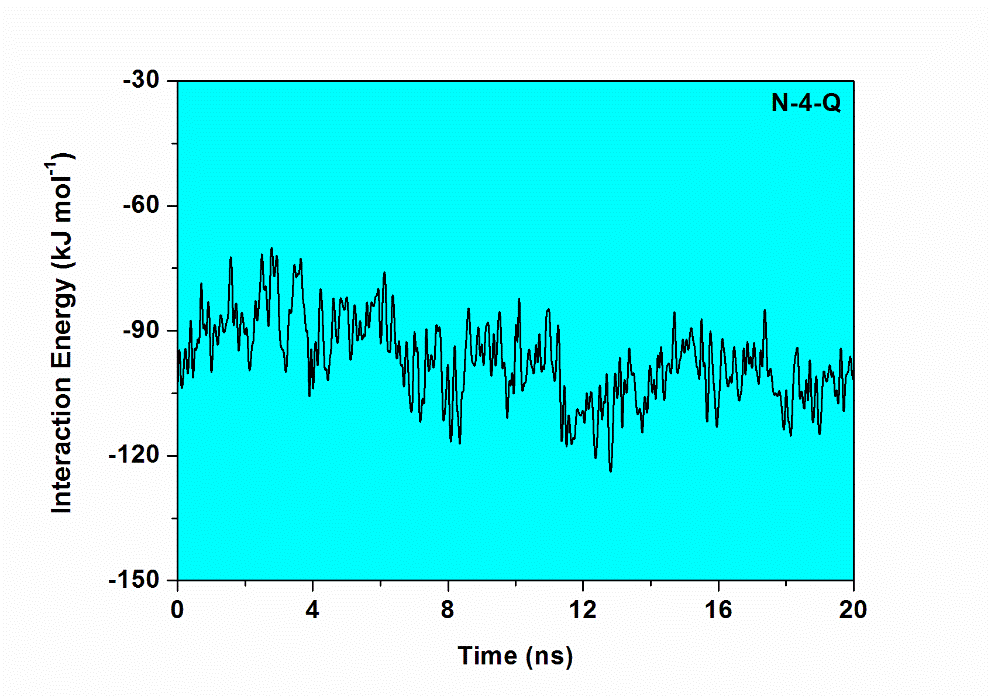

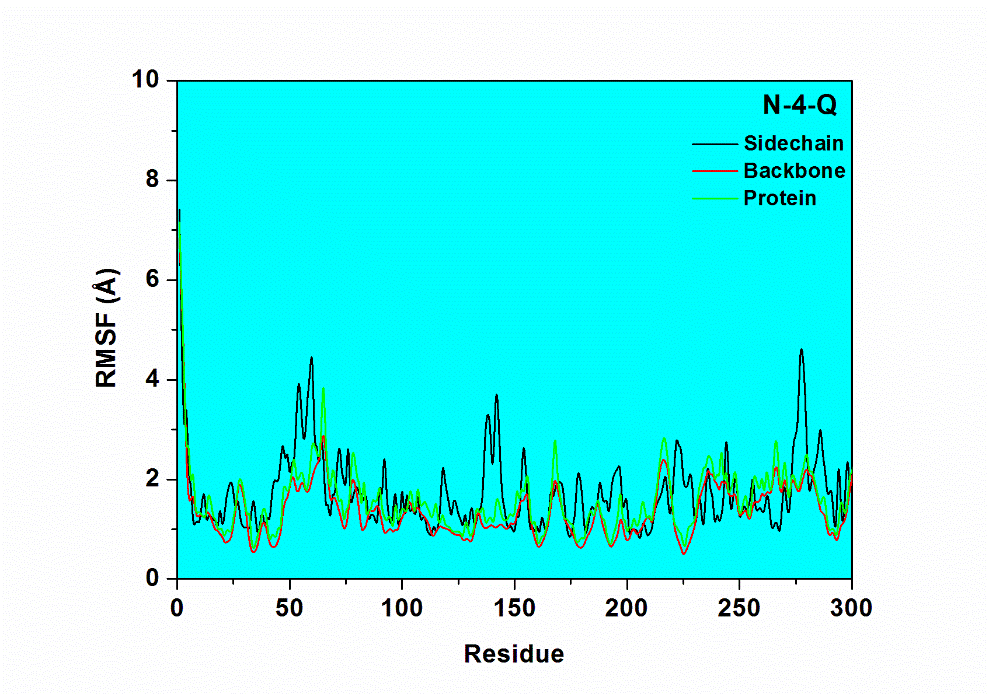


**Figure S4**. RMSD, Total Energy, Hydrogen Bond, Interaction Energy and RMSF graphs of a 20 ns simulation of the N-4-Q.


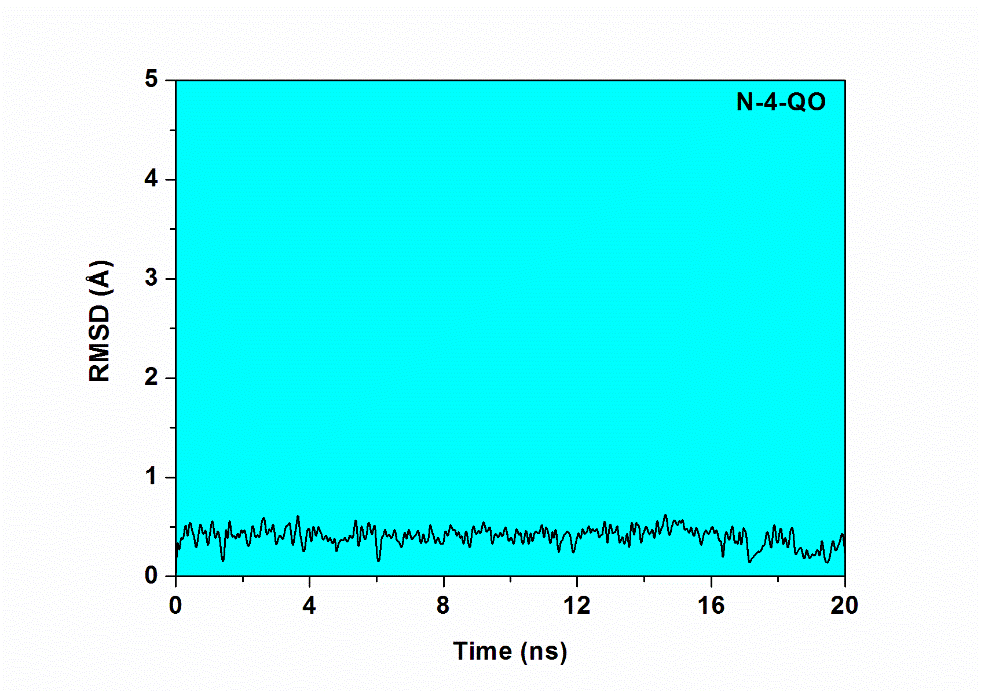

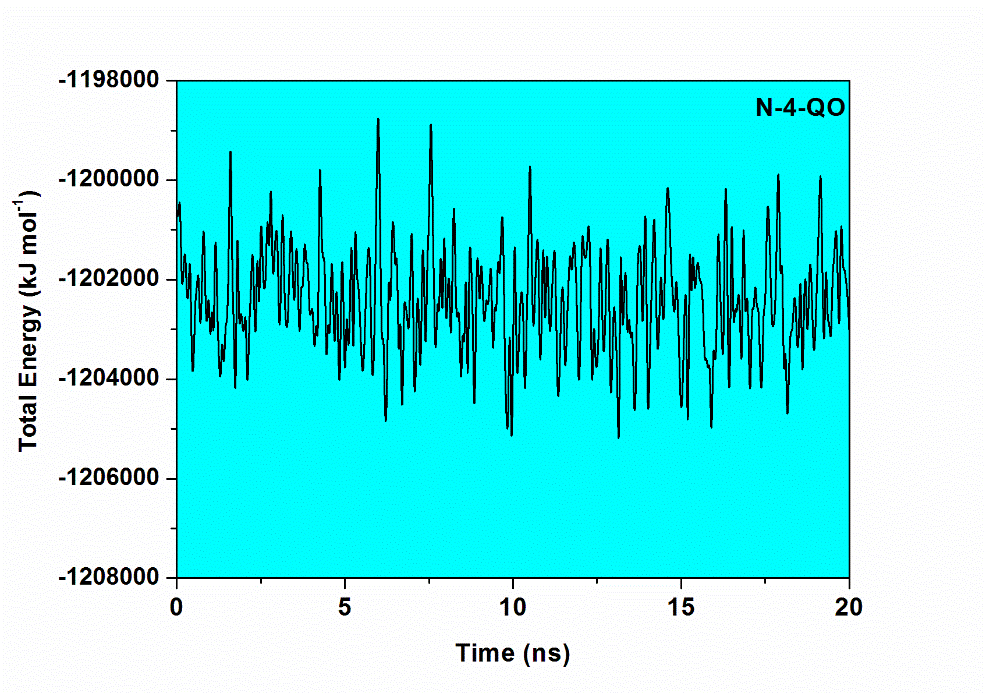

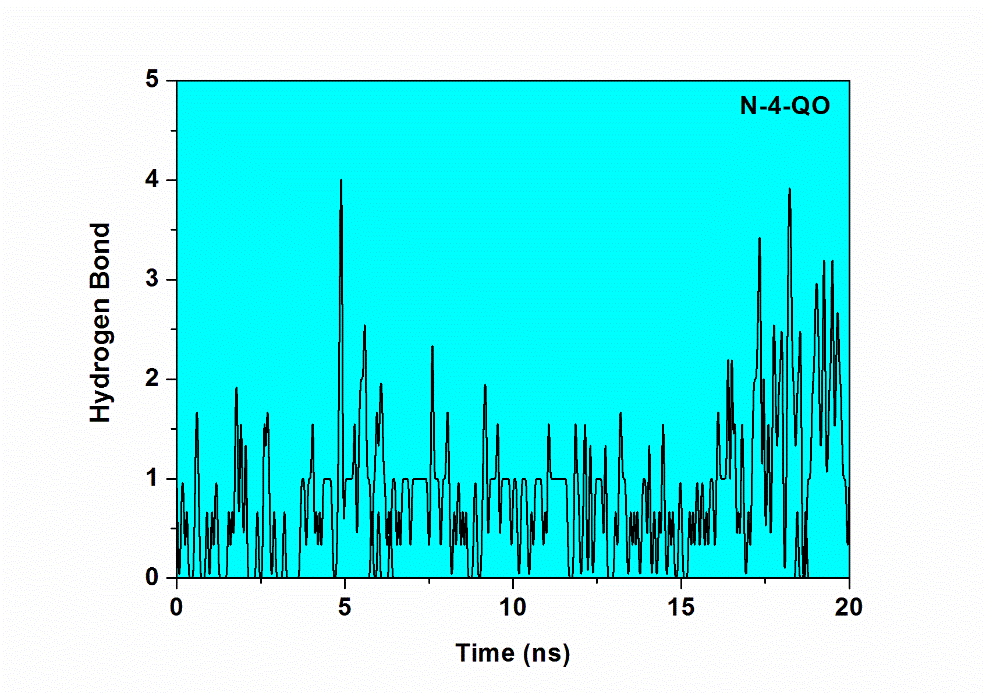

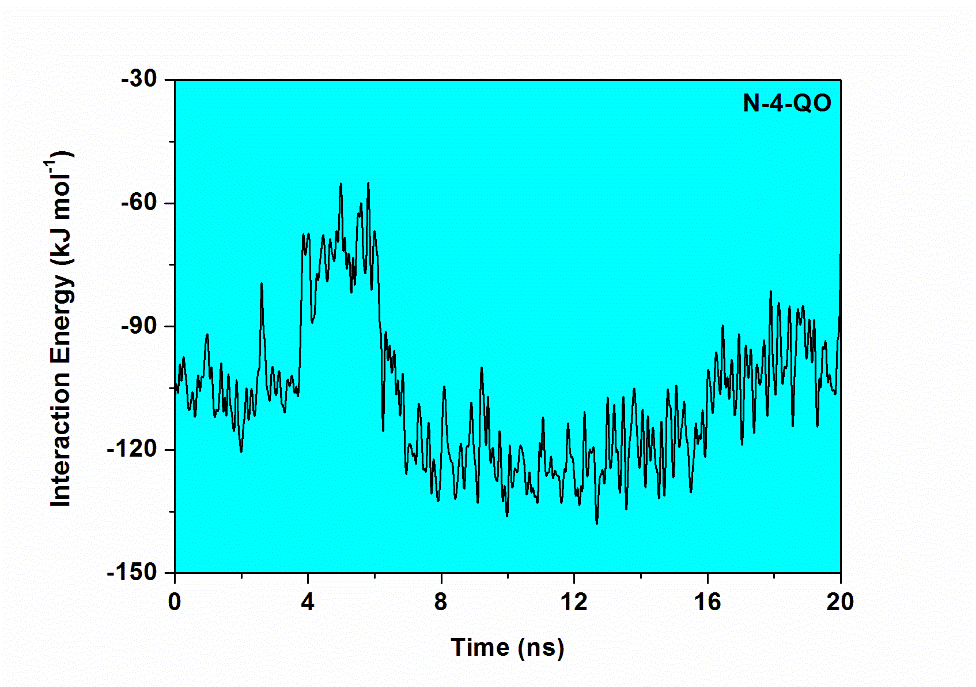

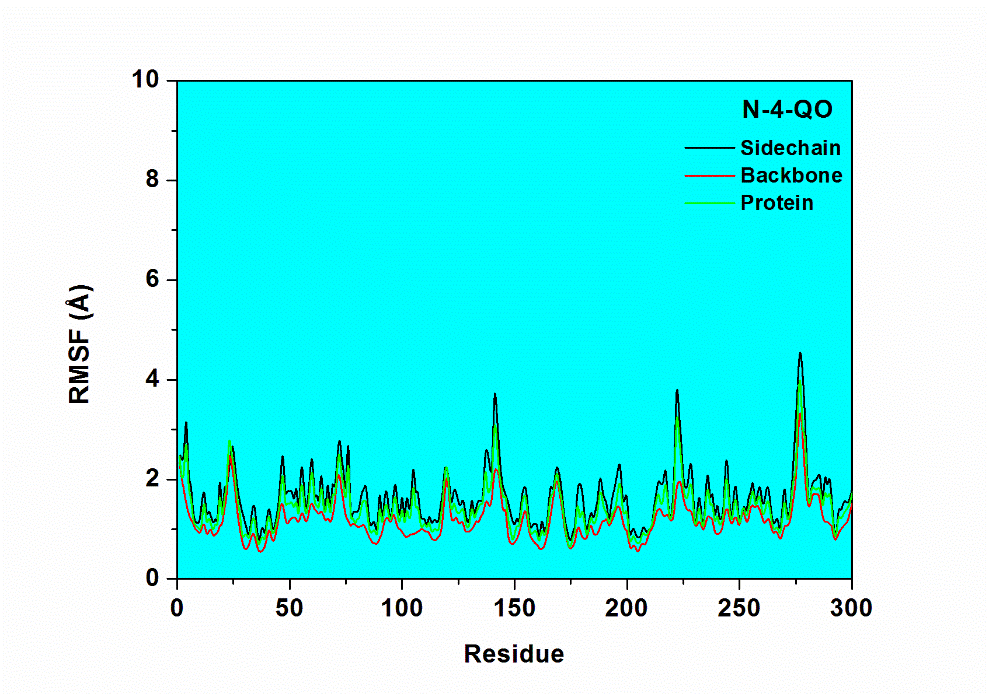


**Figure S5**. RMSD, Total Energy, Hydrogen Bond, Interaction Energy and RMSF graphs of a 20 ns simulation of the N-4-QO.


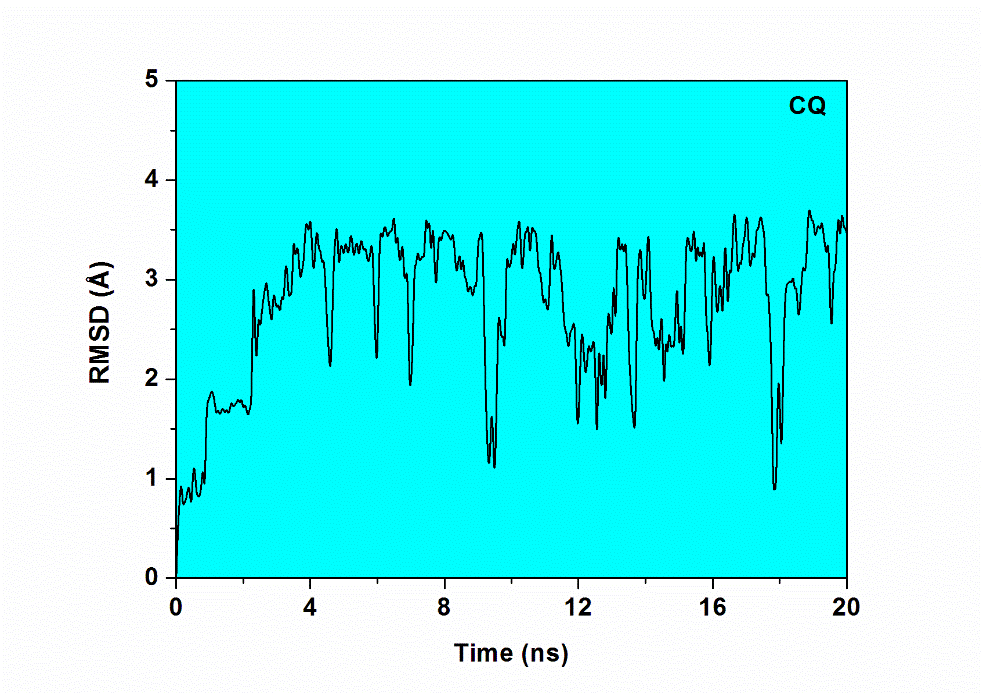

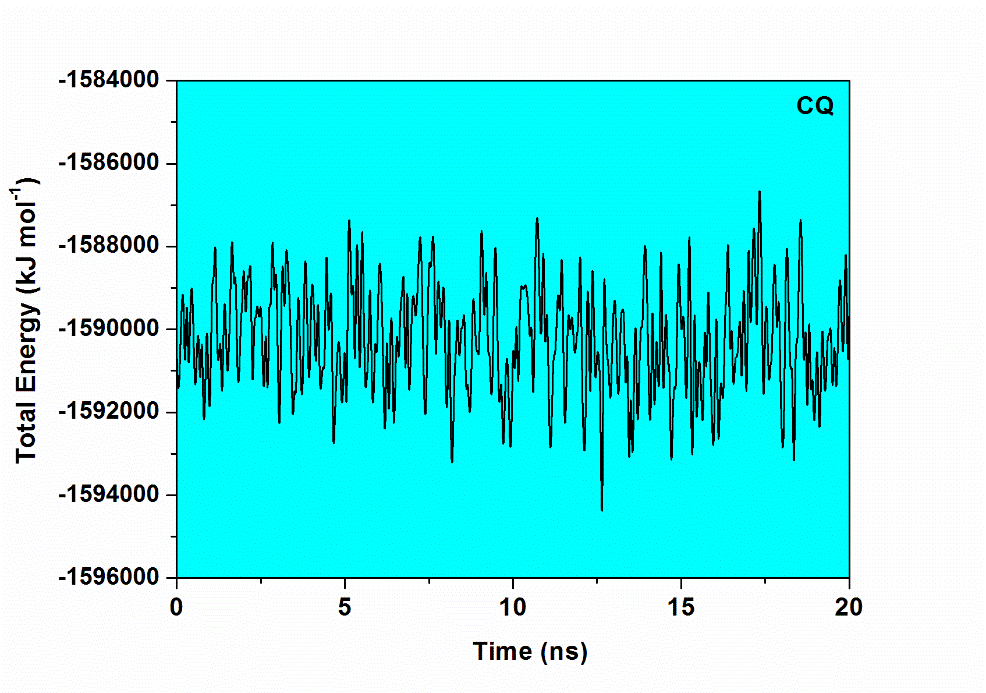

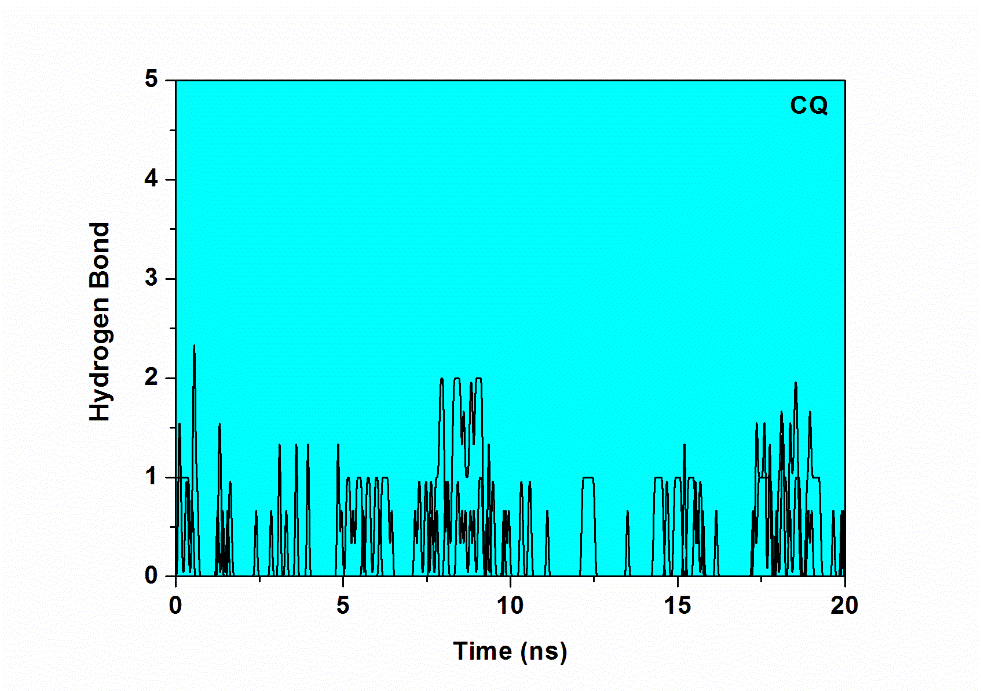

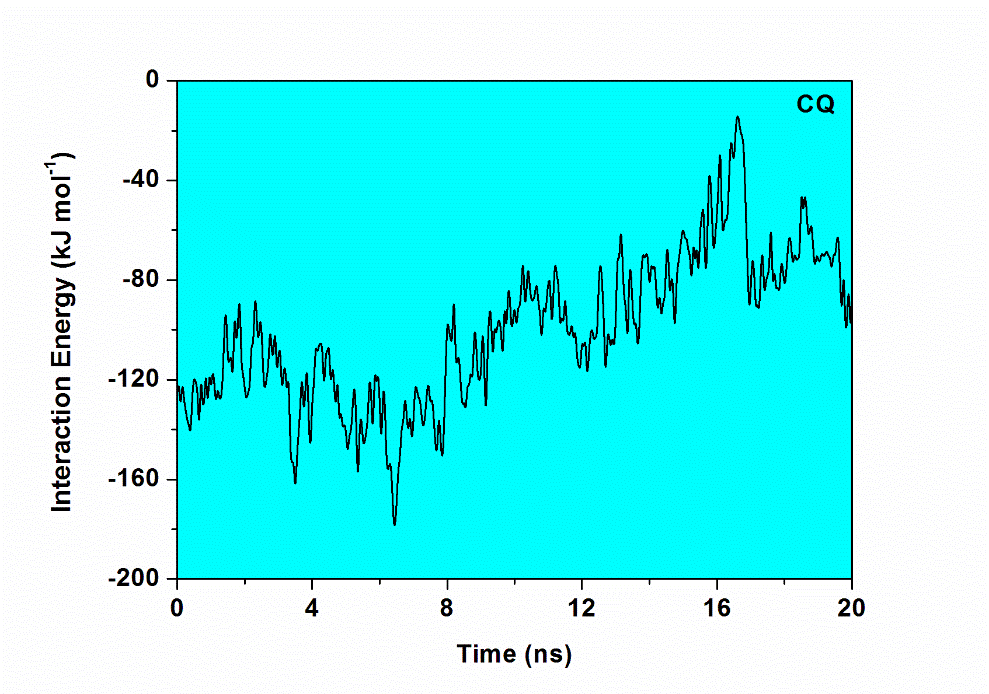

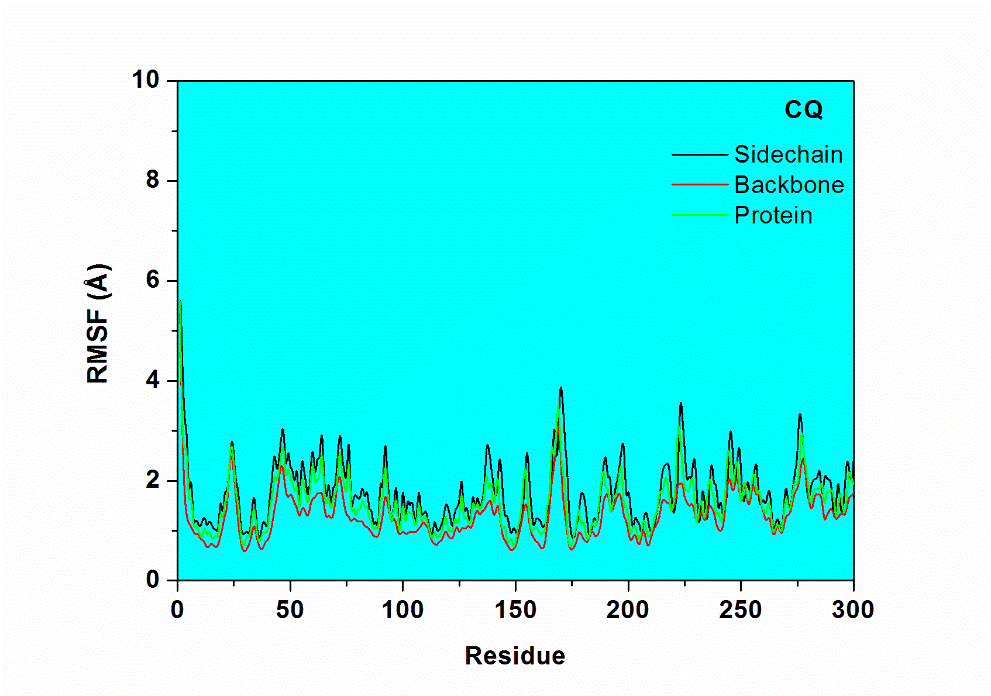


**Figure S6**. RMSD, Total Energy, Hydrogen Bond, Interaction Energy and RMSF graphs of a 20 ns simulation of the CQ.


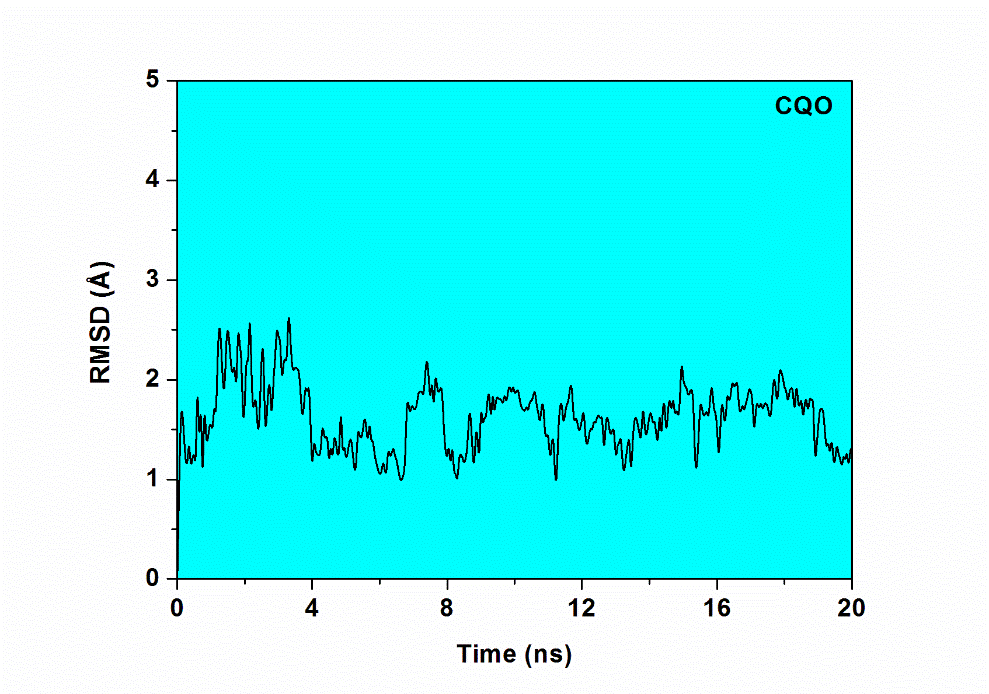

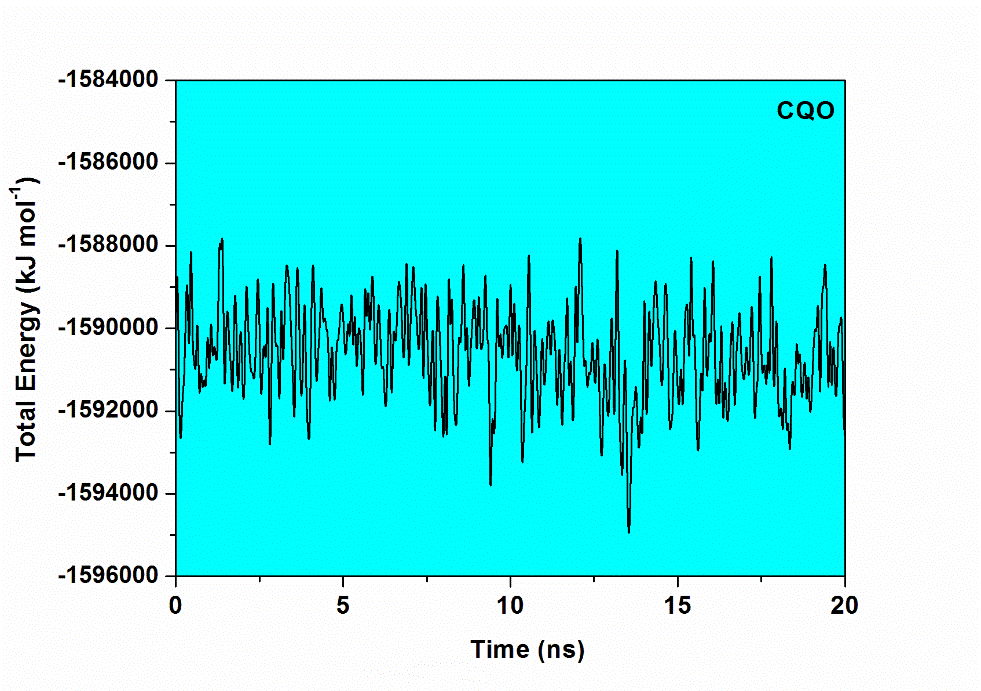

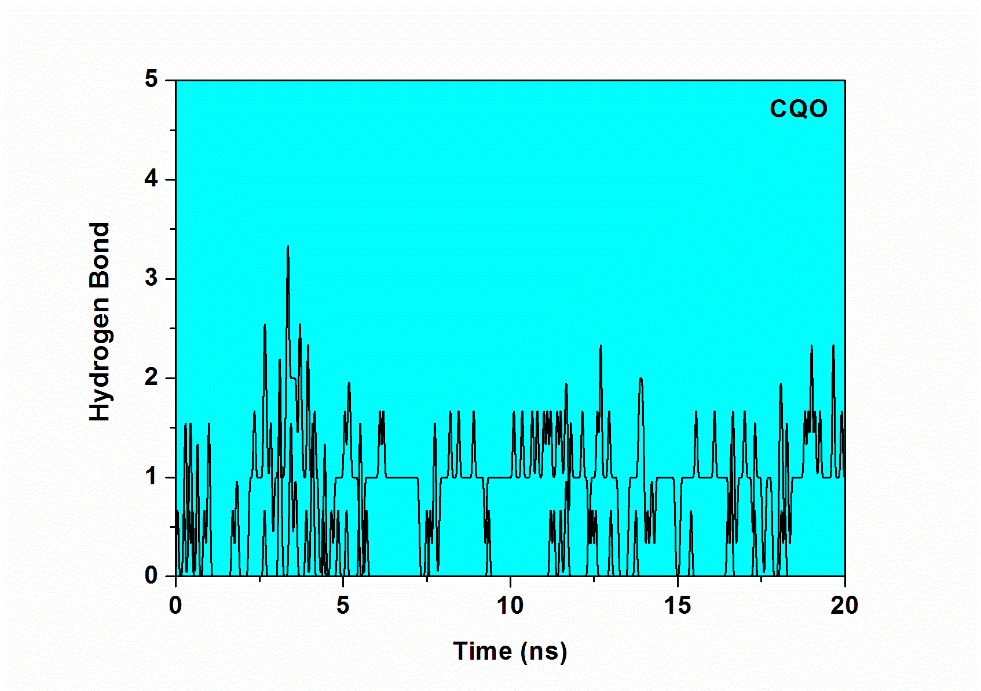

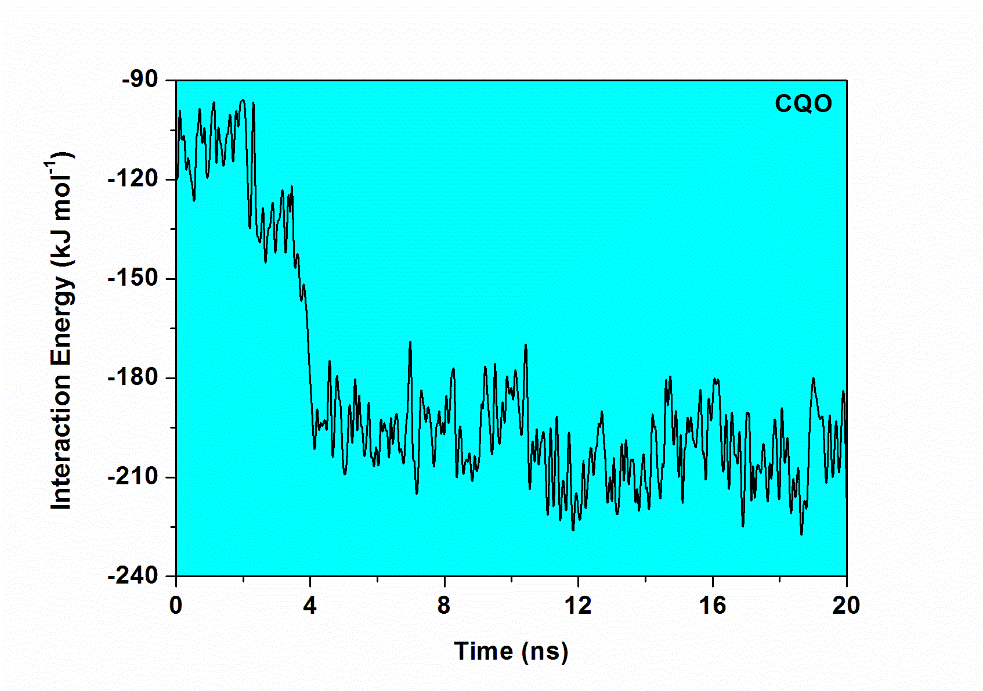

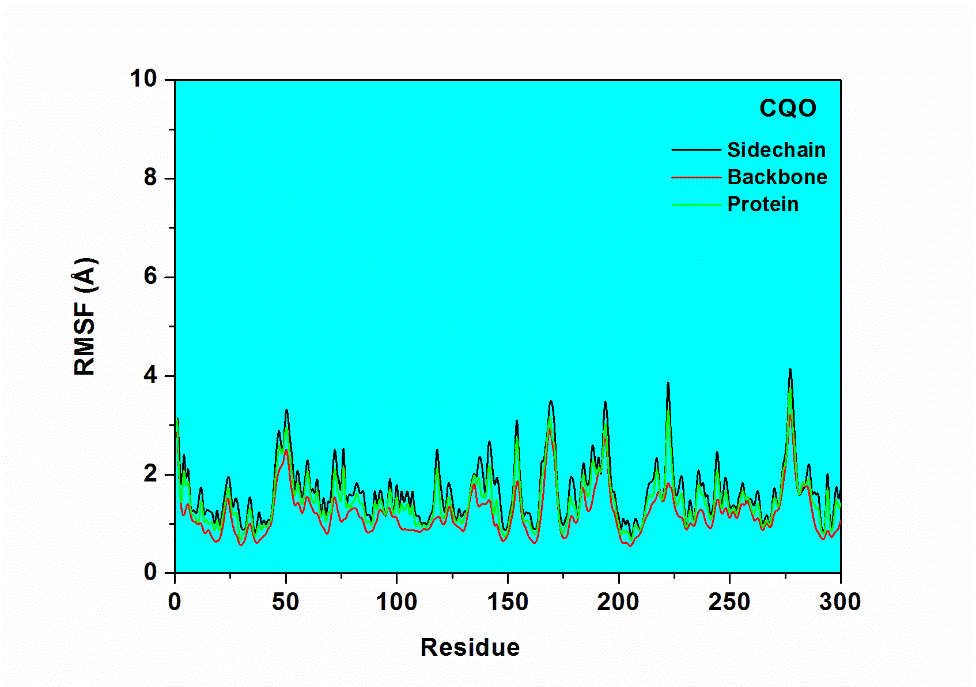


**Figure S7**. RMSD, Total Energy, Hydrogen Bond, Interaction Energy and RMSF graphs of a 20 ns simulation of the CQO.


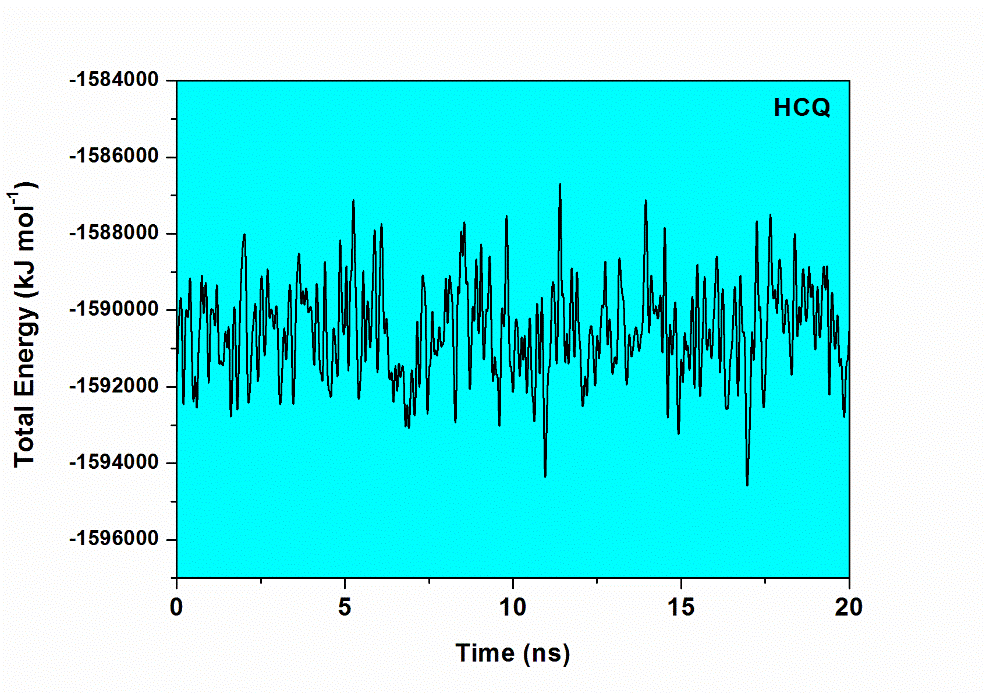

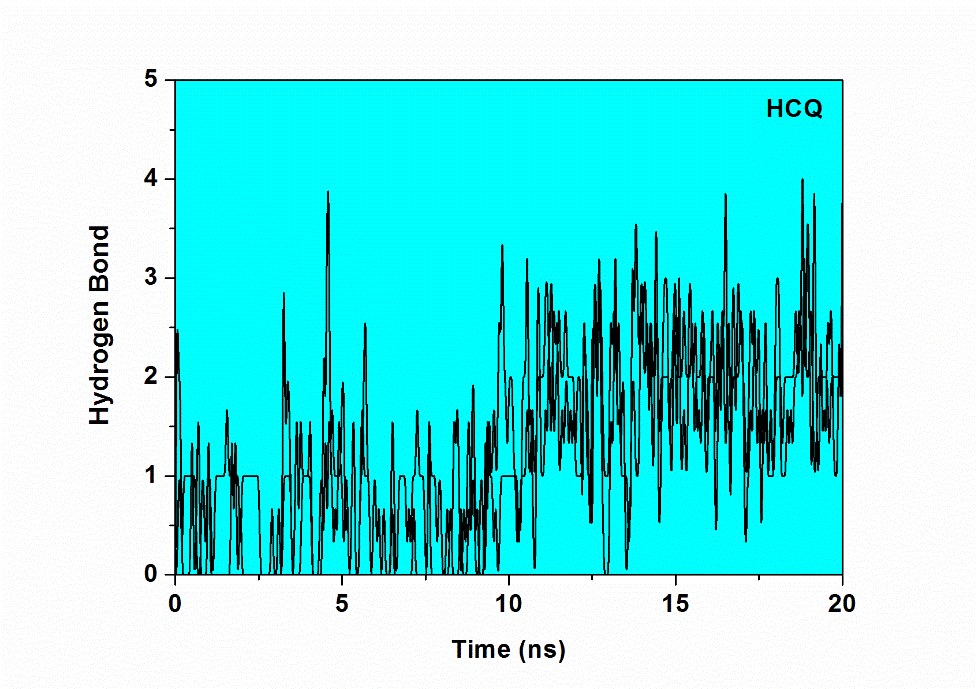

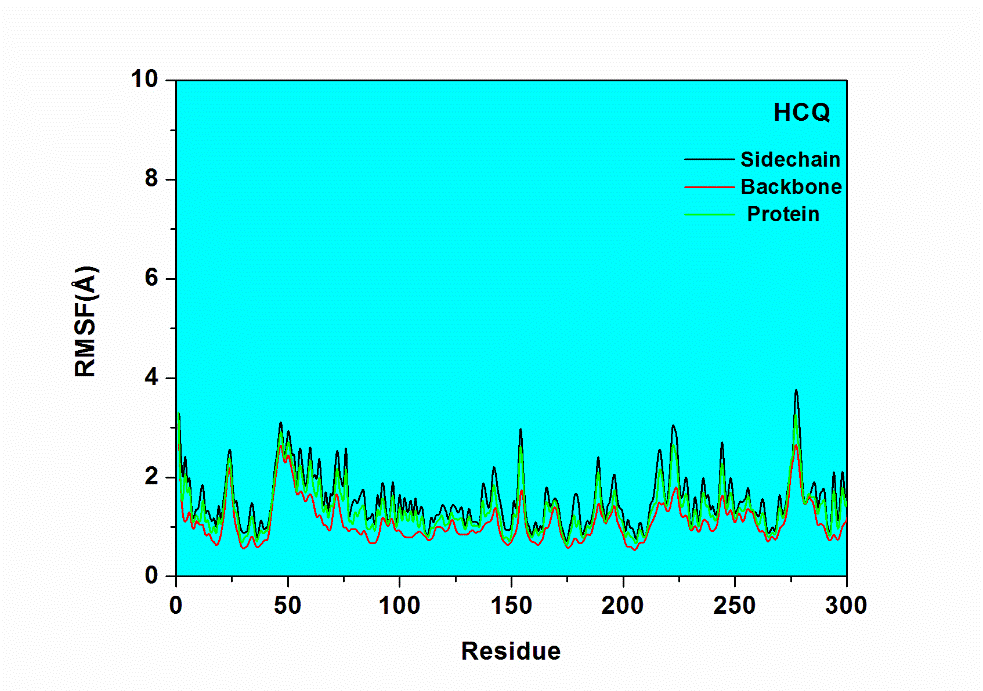

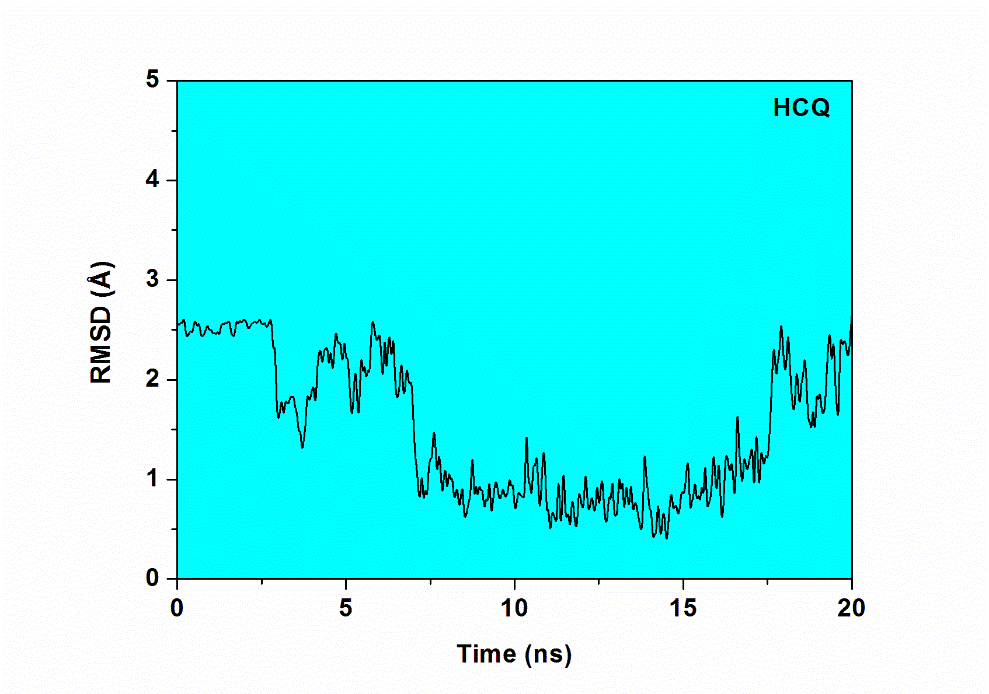

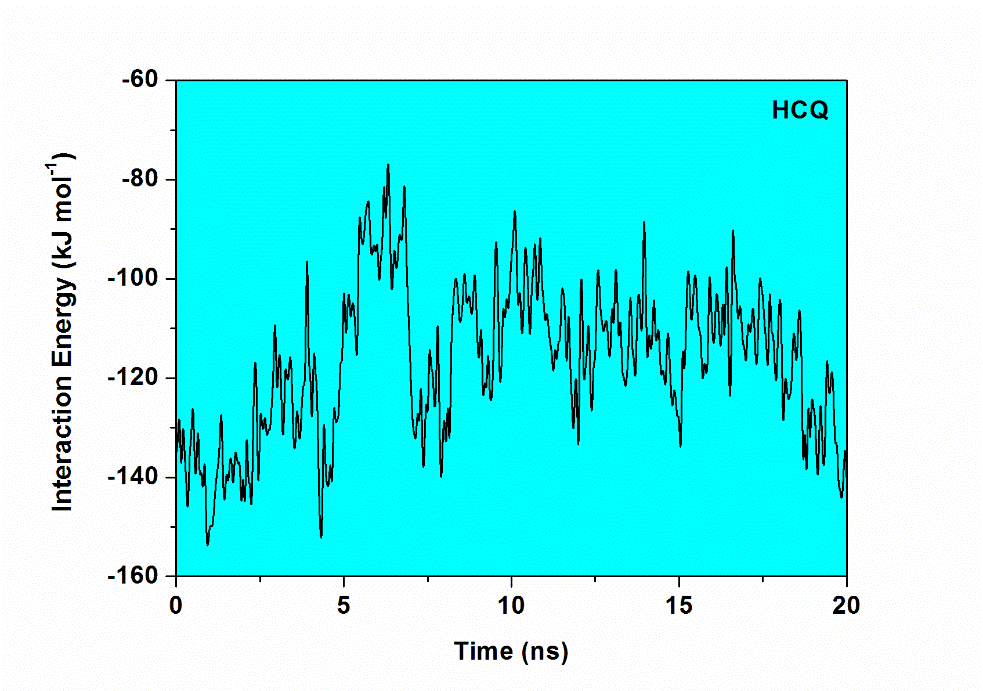


**Figure S8**. RMSD, Total Energy, Hydrogen Bond and Interaction Energy graphs of a 20 ns simulation of the HCQ.


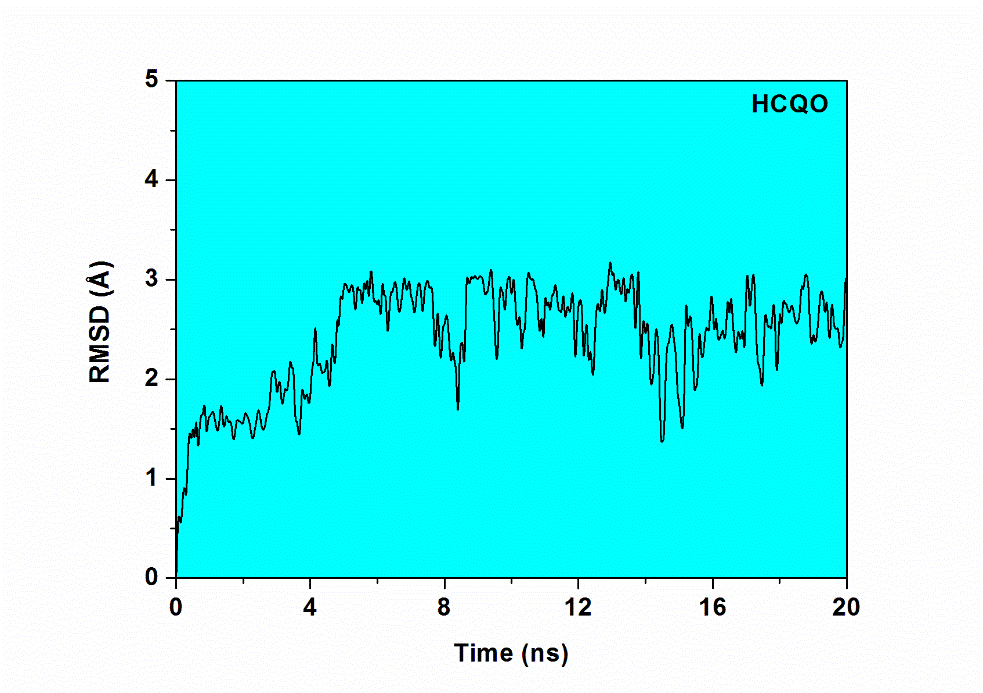

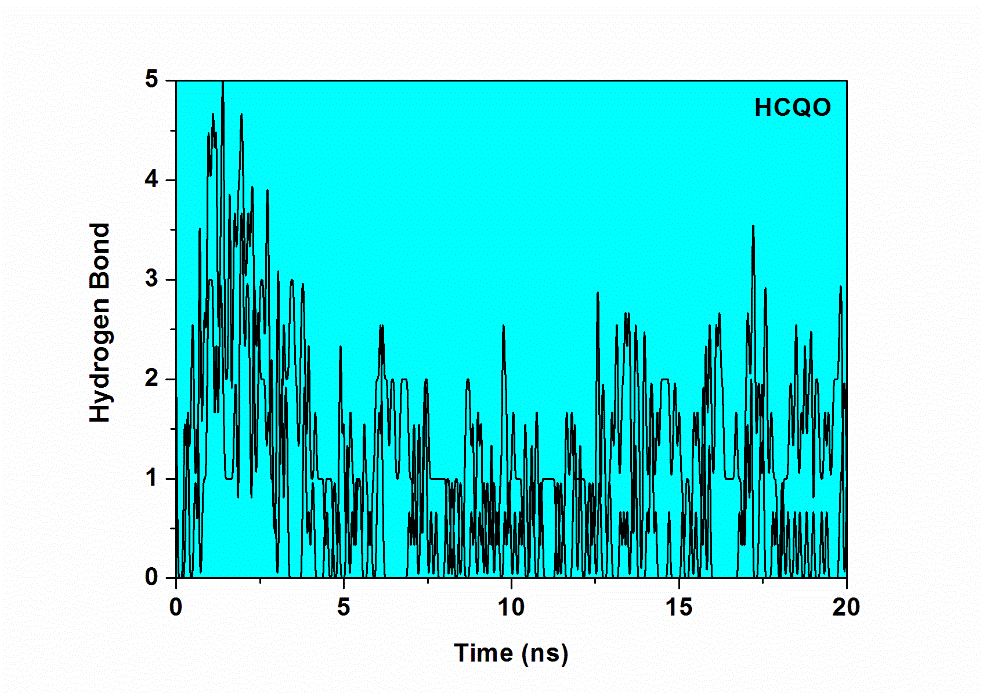

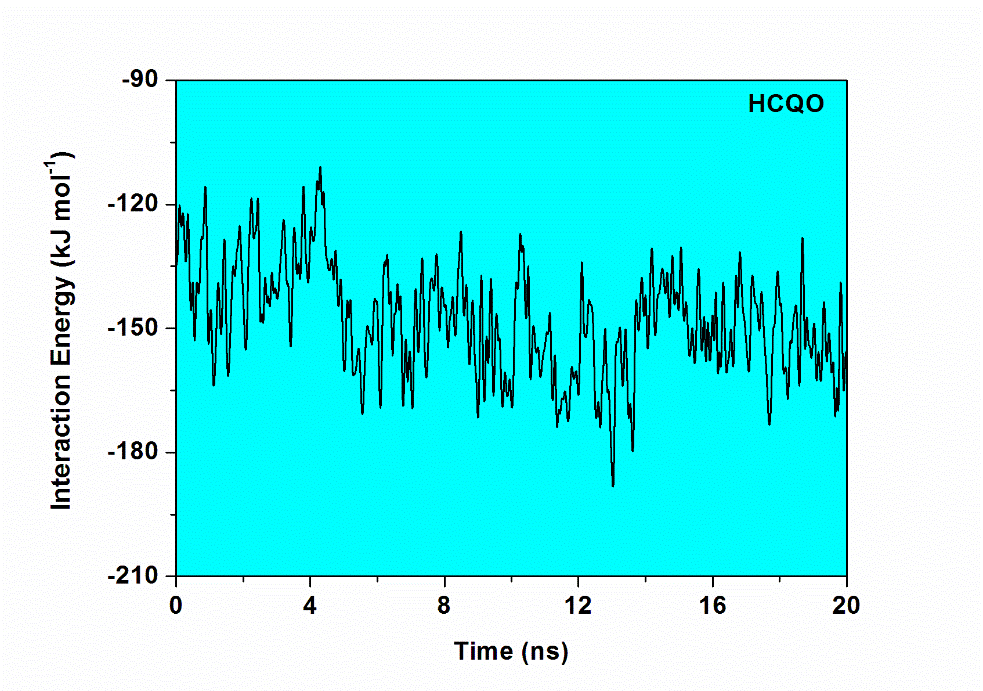

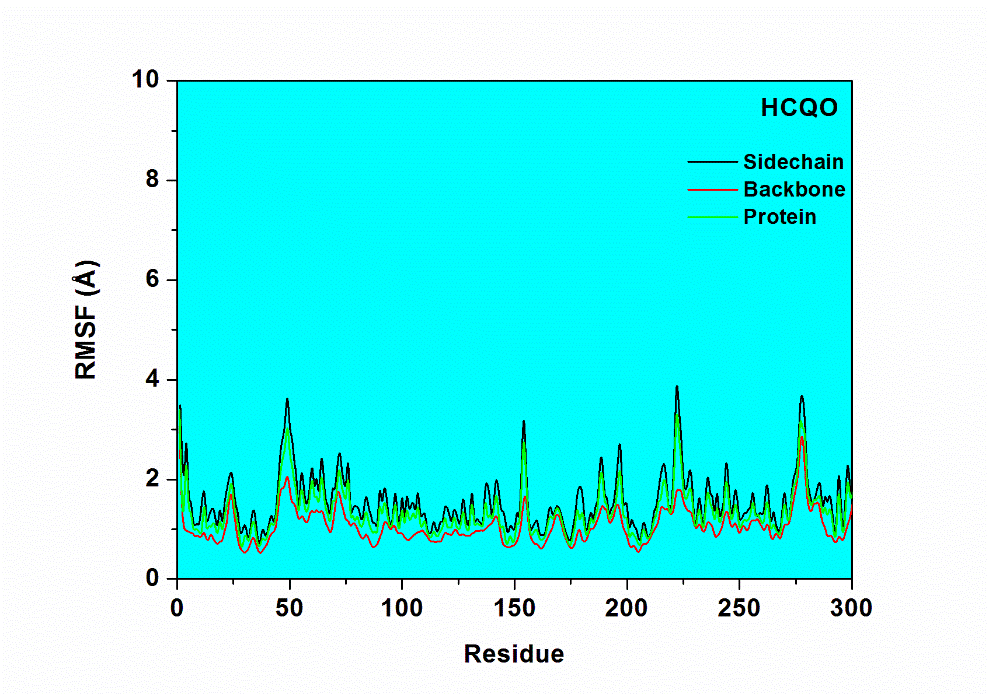

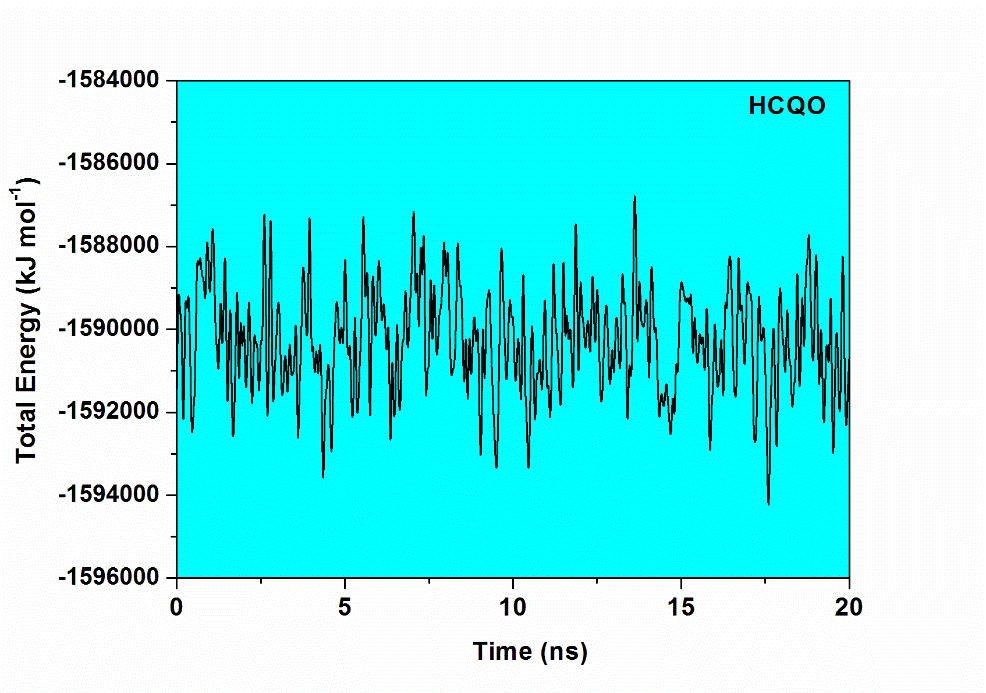


**Figure S9**. RMSD, Total Energy, Hydrogen Bond, Interaction Energy and RMSF graphs of a 20 ns simulation of the HCQO.

**Figure S10**. Chemical structures of the α-ketoamides used in the work.


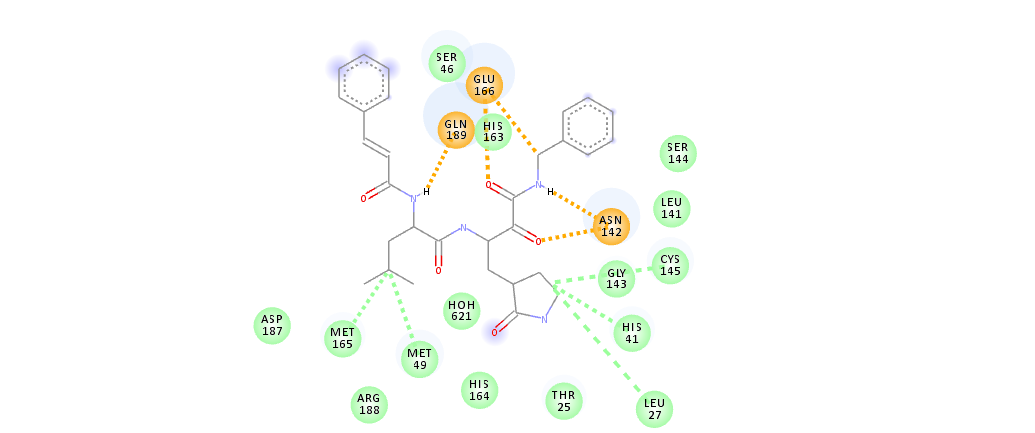


**11n**


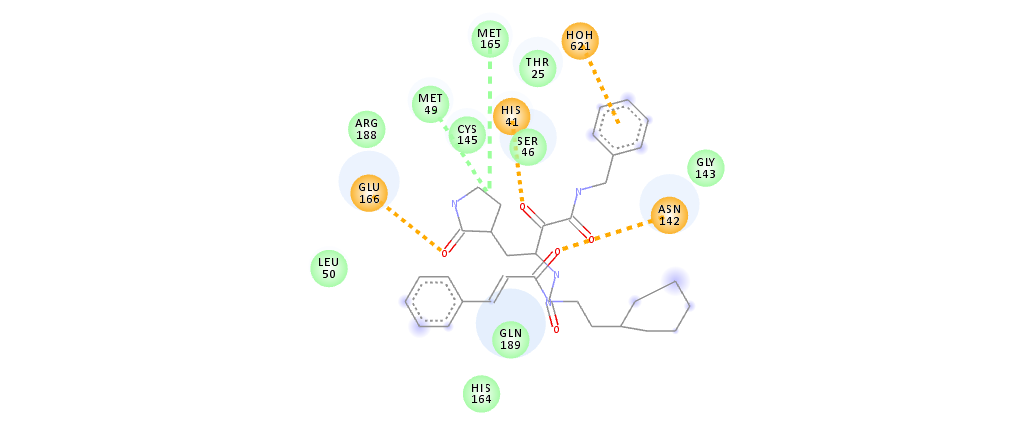

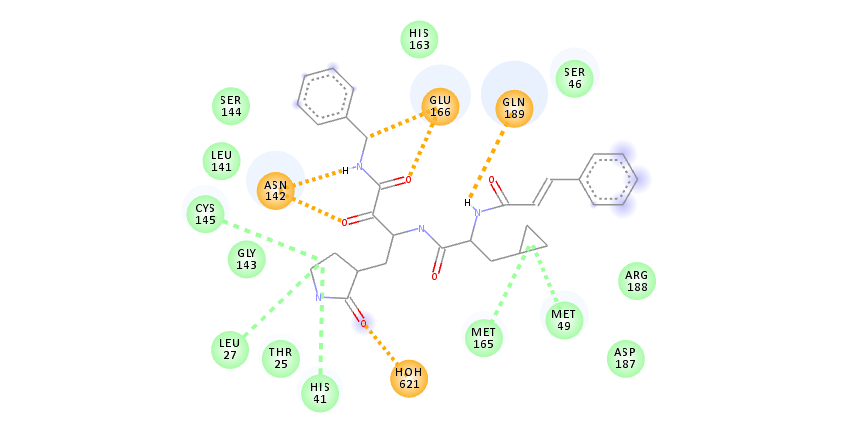


**11r**

**11s**

**Figure S11.** Intermolecular interactions performed by the α-ketoamide inhibitors 11n, 11r and 11s in the M^pro^ active site.







**Figure S12**. General Temperature and Pressure graphs acquired from the equilibration process of the systems.
